# Supplementary material for: Glycogen Synthase Kinase 3 Inactivation Drives T-bet-Mediated Downregulation of Co-receptor PD-1 to Enhance CD8+ Cytolytic T Cell Responses
Source: Immunity. 2016 Feb 16;44(2):274–86. doi: 10.1016/j.immuni.2016.01.018 (PMC4760122; doi:10.1016/j.immuni.2016.01.018)
Supplement: Document S1. Figures S1–S5 and Supplemental Experimental Procedures [file mmc1.pdf]

Immunity, Volume *44*

## Supplemental Information

# Glycogen Synthase Kinase 3 Inactivation Drives T-bet-Mediated Downregulation of Co-receptor PD-1 to Enhance CD8$^{+}$ Cytolytic T Cell Responses

**Alison Taylor, James A. Harker, Kittiphat Chanthong, Philip G. Stevenson, Elina I. Zuniga, and Christopher E. Rudd**

**Figure S1**

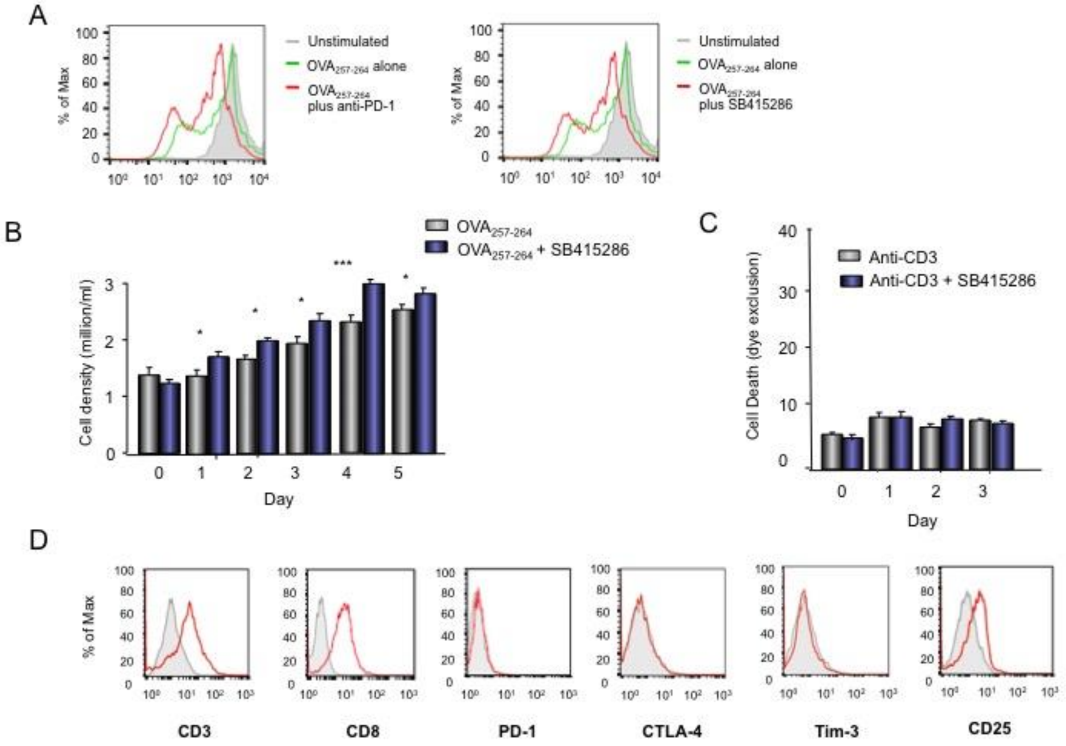


**Figure S1, related to Figure 1. GSK-3 inhibition increased T-cell proliferation and cell number.**

Flow cytometry profiles of OT-1 T-cells activated by EL-4-OVA in the presence or absence of anti-PD-1 or SB415286. OT-1 cells were labelled with CFSE prior to culturing with mitomycin C treated EL-4-OVA cells in the presence or absence of SB415286 or anti-PD1 for 5 days prior to analysis by FACs. Left panel: Profile in the presence or absence of anti-PD-1. Right panel: Profile in the presence or absence of anti-PD-1. The addition of SB415286 enhances this proliferation (red line).

(A) Histogram showing an increase in OT-1 cell number in the presence of SB415286. OT-1 cells were cultured with mitomycin C treated EL-4-OVA cells in the presence or absence of SB415286 or anti-PD1 for 5 days prior to analysis by FACs.

(B) Histogram showing no effect of SB415286 on T-cell viability in response to anti-CD3. OT-1 cells were cultured in the presence of soluble anti-CD3 (2C11) and SB415286

(10μM) for various days. Aliquots of cells were then measured for viability using trypan blue inclusion.

(C) FACs profiles resting OT-1 T-cells showing lack of expression of PD-1, CTLA-4 and Tim-3. Cells were positive for CD3 and CD8.

## Figure S2

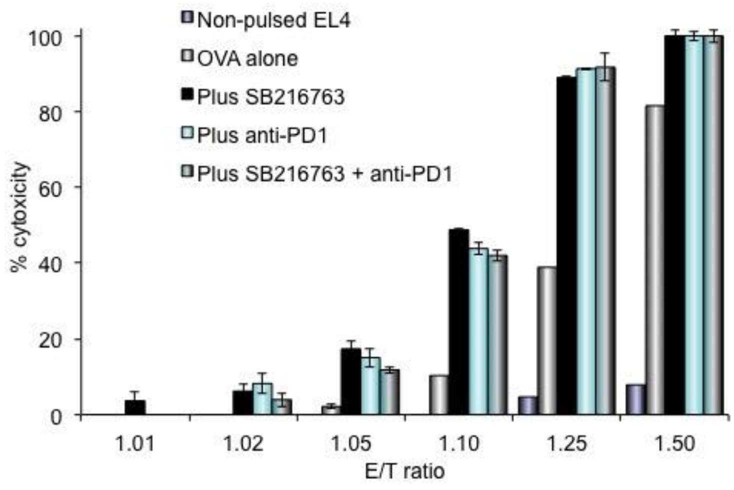


**Figure S2, related to Figure 2. GSK-3 potentiates OT-1 cytolytic killing of EL4-OVA target cells by down-regulating PD-1. Related to Figure 2.**

As in Figure 2 except that the figure shows a measurement of % target killing of EL4-OVA targets by OT-1 CD8+ CTL incubated in the presence or absence of GK-3 inhibitor SB216763 with or without blocking anti-PD-1.

## Figure S3

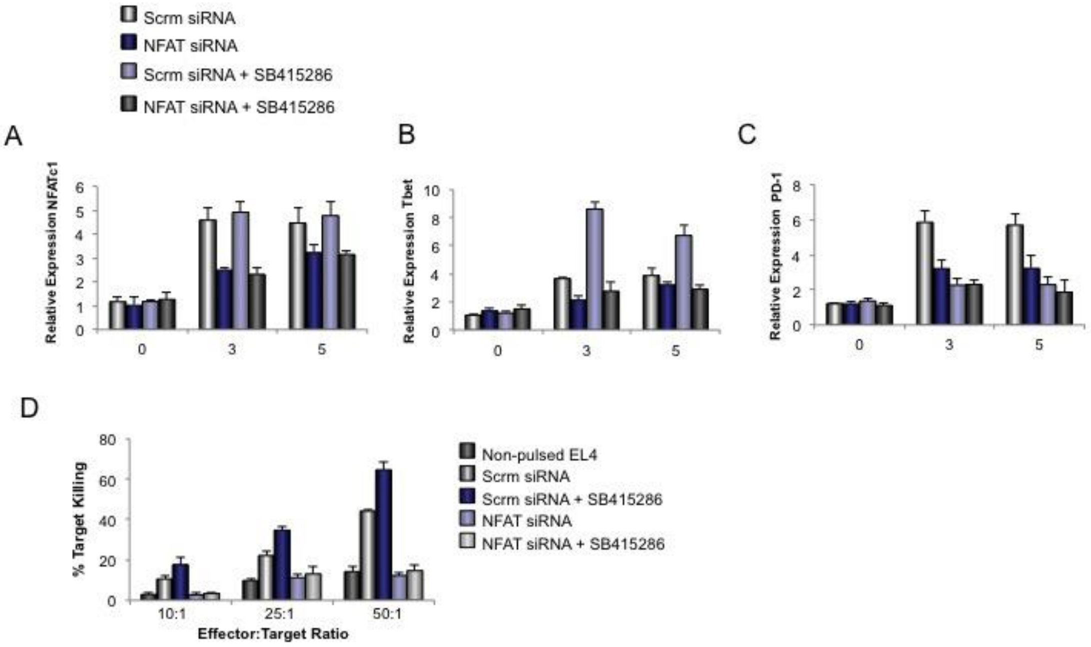


**Figure S3, related to Figure 3. siRNA Knockdown of NFAT inhibits Tbet, PD-1 transcription and CTL function.** OT-1 cells were transfected with either scrambled or NFATc1 siRNA prior to culturing with EL-4-OVA in the presence or absence of SB415286 and assessed for NFATc1 transcription at days 0, 3 and 5 or CTL killing at day 5.

(A) NFATc1 siRNA reduced levels of detectable NFATc1 transcriptions.

(B) NFATc1 siRNA reduced levels of Tbet transcription in the absence or presence of SB415286.

(C) NFATc1 siRNA reduced levels of PD-1 transcription in the absence or presence of SB415286.

(D) NFATc1 siRNA inhibited CTL function when compared to scrambled control siRNA transfected cells.

**Figure S4**

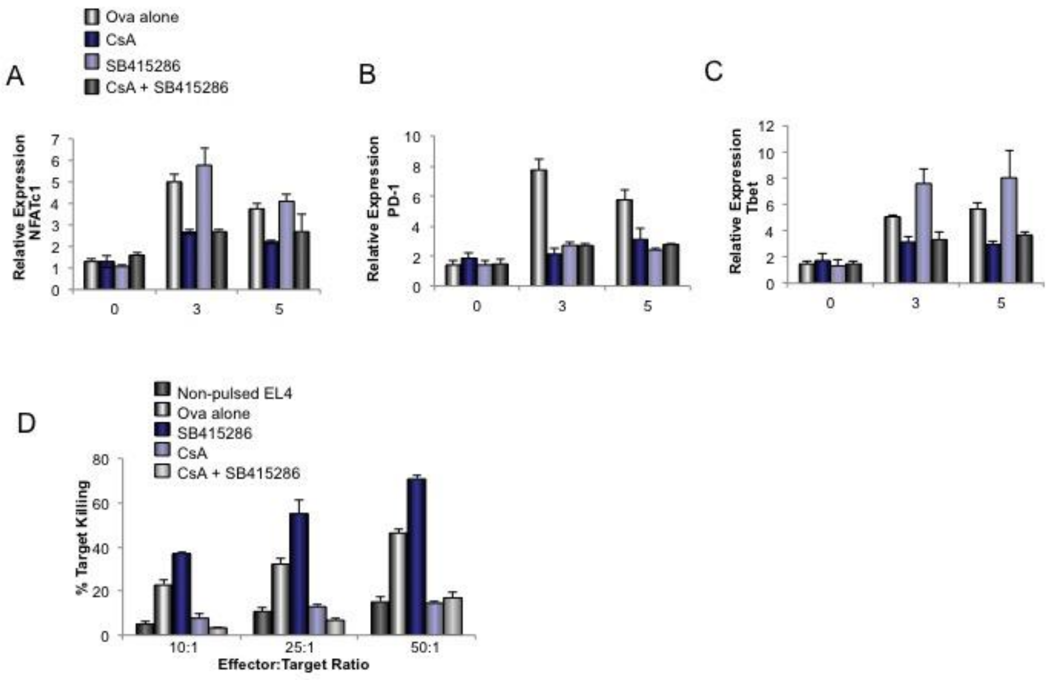


**Figure S4, related to Figure 3. Inhibition of NFAT by Cyclosporin A (CsA) reduces PD-1 and Tbet expression and cytolytic activity.** OT-1 cells cultured with EL-4-OVA and cyclosporine A (CsA) in the presence or absence of SB415286 and assessed for transcription at days 0, 3 and 5 or CTL killing at day 5.

(A) CsA reduced levels of detectable NFATc1 transcriptions.

(B) CsA reduced Tbet transcription in the absence or presence of SB415286.

(C) CsA reduced PD-1 transcription in the absence or presence of SB415286.

(D) CsA siRNA inhibited CTL function when compared to scrambled control siRNA transfected cells.

Figure S5

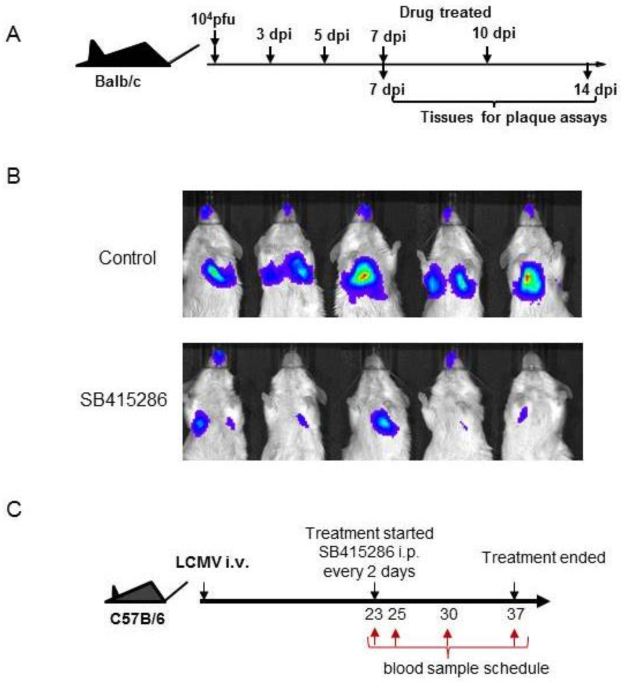


**Figure S5, related to Figures 5 and 6. *In vivo* GSK-3 inhibition increased acute MHV-68 viral clearance via PD-1 down-regulation.** Balb/c mice were intra-nasally infected with MHV-68 with/without an intra-peritoneal injection of SB415286. **(A**) Depicts scheme of injection regime in Figure 5. **(B)** Images from luciferase imaging of mice on 7-day post-infection (upper panel) non-treated; (lower panel) SB415286 in Figure 5. (**C**) Depicts scheme of injection regime for LCMV-Cl 13 model in Figure 6.

## EXTENDED EXPERIMENTAL PROCEDURES

Mice

C57BL/6 and BALB/c (Harlan UK Ltd) were housed at the Department of Pathology and the Central Biological Services (Cambridge University). Mice infected when 6–8 weeks old under the Home Office Project Licence 80/2189 (obtained from The Jackson Laboratory).

### Antibodies/Reagents

Anti-CD3 (2C11; BioXCell), anti-PD-1 (CD279, J43; BioXCell), anti-CTLA-4 (9H10 BioXCell) and PD-L1 (E1L3N; Cell Signaling Technology), anti-Granzyme B (GMZB; ab 17929; Abcam plc), anti-T-bet / Tbx21 (ab53174; Abcam plc) and anti-GSK-3α/β (Biolegend) were purchased commercially. Similarly, various antibodies to CD44, CD62L, Tim3, BTLA, NKG2D, CD122, IL-2R alpha, CD25, FasL and CD8 were purchased from Cell Signaling Technology, Abcam plc and Biolegend as directly conjugated antibodies. Peptide 10nM $OVA_{257-264}$ was purchased from Bachem, while biotinylated $D^b$ $GP_{33-41}$ monomers were provide by the NIH tetramer facility and APC labelled tetramers generated using Streptavidin-APC (Invitrogen). siRNAs for GSK-3α/β, GSK-3α and Tbet and control scrambled siRNAs were synthesized by Cell Signaling Technology.

GSK3 inhibitors were purchased from various sources including SB415286 3-(3-chloro-4-hydroxyphenylamino)-4-(2-nitrophenyl)-1H-pyrrole-2,5-dione and SB216763 [3-(2,4-dichlorophenyl)-4-(1-methyl-1H-indol-3-yl)-1H-pyrrole-2,5-dione] from Abcam plc; CHIR-99021 (CT99021) [6-(2-(4-(2,4-dichlorophenyl)-5-(4-methyl-1H-imidazol-2-yl)pyrimidin-2-ylamino)ethylamino)nicotinonitrile hydrochloride] and L803-mts from T*ocris* Bioscience *Boston* Biochem, R & D systems.

### Cell Culture and Nuclear Transfection

Primary mouse T cells (OT-1, C57/b6) were isolated from spleens and cultured *in vitro* in RPMI 1640 medium supplemented with 10% FCS, 50 μM β-mercaptoethanol, 2 mM L-glutamine, 100 U/ml penicillin and streptomycin, (GIBCO)(Raab et al., 2010; Veale et al., 1999). Spleen cells were treated with a hypotonic buffer with 0.15M NH4CL, 10mM KHCO3 and 0.1mM EDTA, pH 7.2 to eliminate red blood cells. A T cell enriched population was purified by use of T cell purification columns (R&D Systems, Minneapolis, MN). OVA specific CD8+ cytoytic T-cells were generated by incubating isolated splenocytes OT-1 mice with SIINFEKL peptide of OVA ($OVA_{257-264}$) at 10ng/mL for 5-7 days. Alternatively, the thymoma EL-4 cell line was used to present $OVA_{257-264}$ to primary T-cells. EL-4 cells were incubated with 10nM $OVA_{257-264}$ peptide (Bachem) for 1 hour at 37°C and treated with mytomycin C (Sigma-Aldrich, St. Louis, MO) (final concentration of 10μg/mL) prior to mixing with primary T-cells by co-culturing at a ratio of 1:5 of EL4 and T cells in order to generate cytotoxic T cells.

CTLs were generated in the presence or absence of inhibitors and/or anti-PD-1 or anti-PDL-1 blockade for 5-7 days prior to washing and analysis by FACs, PCR or in cytoxicity assays. In some cases, naïve OT-1 T cells were first incubated with OVA peptide with or without GSK-3 inhibitors and/or anti-PD-1 or PD-L1-Fc (1-3ug/ml). All compounds were suspended in DMSO to give a stock solution of 25mM and diluted to a concentration of 1-10uM *in vitro*. NSC 87877 (inhibitor of SHP1/2 PTPs and dual-specificity phosphatase 26 (DUSP26), used as a concentration of 50μM (T*ocris* Bioscience *Boston* Biochem) (Song et al., 2009) and cyclosporin A (CsA) (Sigma) at 0.5ug/ml.

In certain cases, naïve cells were subjected to nuclear transfection in the presence of various siRNA oligos (i.e. GSK-3). 3.0-5.0ug of siRNAs were added to 1 x $10^6$ T-cells and suspended in 100ul of Nucleofector™ solution for T cells (Amaxa Biosystems, Cologne, Germany). Cells and oligos were then transferred into a cuvette and electroporated using program X-01 of the Nucleofector™ (Amaxa

Biosystems), and then immediately transferred into pre-warmed RPMI medium, as previously described(Smith et al., 2013; Valk et al., 2006). In certain instances, pre-activated T-cells were transfected with mouse PD-1 in an SR alpha promoter (pPD1) composed of the simian virus 40 early promoter and the R-U5 segment of human T-cell leukemia virus type 1 long terminal repeat (Takebe et al., 1988).

**Cytotoxicity Assays**

For *in vitro* cytotoxic assays, transfected T cells were plated in 96-well plates at the start of culture with activating EL4 cells pulsed with OVA peptide except in the case of the MHV-68 in which EL4s were pulsed with the M2 peptide ($M2_{91–99}$, GFNKLRSTL) (SIGMA-Genosys, Haverhill, UK) (Husain et al., 1999). Cytotoxicity was assayed using a Cytotox 96 nonradioactive kit (Promega) following the instructions provided. In brief, purified T cells were plated in 96-well plates at the effector/target ratios shown using $10^4$ EL4 (OVA peptide-pulsed, except in the case of the MHV-68 in which EL4s were pulsed with the M2 peptide. Target cells per well were in a final volume of 200 µl per well using RPMI lacking phenol red. Lactate dehydrogenase release was assayed after 4 h incubation at 37°C by removal of 50 µl supernatant from each well and incubation with substrate provided for 30 min and the absorbance read at 490 nm using the Thermomax plate reader (Molecular Devices). Percentage cytotoxicity = ((experimental $\text{effector}_{\text{spontaneous}}$ – target spontaneous)/($\text{target}_{\text{maximum}}$ – target spontaneous)) x 100. All cytotoxicity assays were reproducible in at least three independent assays (Jenkins, MR *et al*, 2009). Scatchard analysis was conducted based on the ratio of dead target cells to live effector cells versus the total number of targets killed. Linearized data obtained first from a non-linear curve displaying E/T ratios in order to determine Kd and BMax values using GraphPad Prism software.

**Quantitative real-time PCR**

Single-strand cDNA was synthesized with an RT-PCR kit (Qiagen, Hilden, Germany) according to the manufacturer's instructions. Reverse transcription was performed using the RNA polymerase chain reaction (PCR) core kit (Applied Biosystems). Quantitative real-time PCR used SYBR green technology (Roche) on cDNA generated from the reverse transcription of purified RNA. After preamplification (95°C for 2 min), the PCRs were amplified for 40 cycles (95°C for 15 s and 60°C for 60 s) in a sequence detection system (PE Prism 7000; Perkin-Elmer Applied Biosystems, USA). The exponential phase, linear phase and plateau phase of PCR amplification were carefully monitored to ensure a measurement of real time transcription (Wacker and Godard, 2005). mRNA expression was normalized against GAPDH expression using the standard curve method. PD-1-FW, 5-CCGCCTTCTGTAATGGTTTGA-3; PD-1-RV, 5-GGGCAGCTGTATGATCTGGAA-3; Tbet-FW, 5-GATCGTCCTGCAGTCTCTCC-3; Tbet-RW, 5-AACTGTGTTCCCGAGGT GTC-3; GAPDH-FW, 5-CAACAGCAACTCCCACTCTTC-3; GAPDH- RW, 5-GGTCCAGGGTT TCTTACTCCTT-3. NFATc1 siRNA was purchased from Santa Cruz (http://www.scbt.com/datasheet-36054-nfatc1-sirna-m.html).

**Chromatin immunoprecipitation (ChIP) assay**

C57/b6 primary T cells were purified using CD3+ T cell enrichment columns, 3 x $10^6$ cells were used as a resting control while remaining cells were cultured with anti-CD3 with/without SB415286 for 72h. Chromatin was prepared from all samples (3 x $10^6$ cells) and used for ChIP assay following the manufacturers protocols - Pierce$^{TM}$ Agarose ChIP kit (Thermo scientific #26156). The immunoprecipitation step was performed independently using one of two T-bet antibodies (H-210 or 4B10, Santa cruz) or a positive control –anti-RNA polymerase II, or negative control Rabbit Ig, both of the latter being provided in the kit. The resulting purified DNA with bound

Ab was then analysed using quantitative RT-PCR. CHiP assay was also performed using Jurkat cell line $3x10^6$ cells with/without SB415286 for 72 hrs

**Luciferase reporter assay**

C57/b6 primary T cells were purified using CD3+ T cell enrichment columns and cultured with anti-CD3 with/without SB415286 for 72h. Cells were washed and transfected with a promoter *Ifng CNS-12* construct driven only by a Tbet element (kind gift of Dr. Graham Lord, King's College), and left to rest overnight. 18h post transfection, cells were incubated in anti-CD3 coated plates ($2x10^5$/well) for 6h at $37^0$C. Plates were washed gently with PBS twice and cells re-suspended in 20ul of Passive lysis buffer (Dual-luciferase® Reporter Assay System (Promega)). Following 20 min incubation on rocking platform, plates incubated at $-20^0$C overnight. Thawed samples transferred to FACs tubes and 50ul of luciferase substrate added before measuring with luminometer, 50ul of Renilla added to each tube and re-measured. Calculations performed using (luciferase measurement/renilla measurement) x 1000.

***In vivo* Priming OT-I Tg cells**

OVA peptide (1μg) was injected intravenously into OT-I Tg mice with and without SB415286 (10μg) in 100μl of PBS. Spleens were harvested after 7 days and T cells purified. Longer experiments utilized a repeat injection on day 7 that was reminiscent of the initial injection. Spleens were then harvested on day 14 and T cells purified.

**Viruses and Luciferase imaging in vivo**

Luciferase tagged- MHV-68 viral stocks were prepared as described (de Lima et al., 2004; Gillet et al., 2006). BALB/c mice were infected with luciferase expressing MHV-68 ($10^4$ p.f.u.) intranasally under general anaesthesia when 6–12 weeks old.

Intranasal infections with anaesthesia were in 30 μl aliquots. Mice underwent a treatment regime as depicted on days 0, 3, 5, 7 and 10 of either PBS or SB415286 100μl intraperitoneally. All experiments conformed to local animal ethics regulations; those in Cambridge also followed Home Office Project Licence 80/2189. For luciferase imaging, mice were injected intraperitoneally with luciferin (2 ug per mouse), anaesthetized with isoflurane and scanned with an IVIS Lumina (Caliper Life Sciences) as previously described (Milho et al., 2009). SB415286 was dosed at 10μg/kg based on a pilot dose response study while anti-PD1/PD-L1 treatment was conducted at 100ug per dose/mouse *in vivo*.

For quantitative comparisons, we used Living Image software (Caliper Life Sciences) to obtain the maximum radiance (photons per s per $cm^2$ per steradian, i.e. photons $s^{-1}$ $cm^{-2}$ $sr^{-1}$) over each region of interest, relative to a negative control region. For viral infectivity assays, MHV-68 stocks were titrated by plaque assay on BHK-21 cells (de Lima et al., 2004). Cell monolayers were incubated with virus (2 h, 37 °C), overlaid with 0.3% carboxymethylcellulose, and 4 days later fixed and stained for plaque counting. Infectious virus in lungs was measured by freeze–thawing the lungs and homogenizing them in 1 ml complete medium prior to plaque assay. Latent virus was measured by infectious centre assay(de Lima et al., 2004): spleen cells were co-cultured with BHK-21 cells, then fixed and stained for plaque counting after 4 days. Plaque assay titres of freeze–thawed lymphoid homogenates were always <1% of infectious centre assay titres, so the latter essentially measured reactivated latent virus.

For LCMV, LCMV-Arm and Cl 13 strains were prepared as described (Harker et al., 2013; Harker et al., 2011). Viruses were also grown, identified, and quantified as described (Ahmed et al., 1984; Borrow et al., 1995). 6- to 8-week-old mice were infected intravenously (i.v.) with $2 \times 10^6$ pfu of LCMV-Arm or Cl 13. Mice received doses of SB415286 or PBS every 48h from day 23 through until day 37. LCMV titers

in the serum were determined by vero cell plaque assays as described previously[53.] Assay vero cells (ATCC) were seeded in 96 well plates and incubated with serial dilutions of serum or tissue homogenate for 20 hr. Cells were fixed with 1% paraformaldehyde, blocked with PBS containing 10% FBS for 1 hr, and then incubated with supernatant from 113 hybridoma cells (kindly provided by M.B. Oldstone, TSRI). Finally, the cells were incubated with goat anti-mouse IgG conjugated with Cy3 (Jackson Immunoresearch) and foci were counted by fluorescent microscope. LCMV-specific ELISAs and avidity assays were done as previously described ( Harker et al., 2011).

**Statistical Analysis**

Unpaired Student's t tests or ANOVA tests were performed using the InStat 3.0 software (GraphPad, CA). In certain instances, statistics were done using 2-way ANOVA, or by non-parametric Mann Whitney at each timepoint. * $P < 0.05$, ** $P < 0.01$, *** $P < 0.001$.

**References**

Ahmed, R., Salmi, A., Butler, L.D., Chiller, J.M., and Oldstone, M.B. (1984). Selection of genetic variants of lymphocytic choriomeningitis virus in spleens of persistently infected mice. Role in suppression of cytotoxic T lymphocyte response and viral persistence. J. Exp. Med. *160*, 521

Borrow, P., Evans, C.F., and Oldstone, M.B. (1995). Virus-induced immunosuppression: immune system-mediated destruction of virus-infected dendritic cells results in generalized immune suppression. Journal of virology *69*, 1059-1070.

de Lima, B.D., May, J.S., and Stevenson, P.G. (2004). Murine gammaherpesvirus 68 lacking gp150 shows defective virion release but establishes normal latency in vivo. Journal of virology *78*, 5103-5112.

Gillet L, Gill MB, Colaco S, Smith CM, Stevenson PG. (2006). Murine gammaherpesvirus-68 glycoprotein B presents a difficult neutralization target to
