# Supplementary material for: Glycogen Synthase Kinase 3 Inactivation Drives T-bet-Mediated Downregulation of Co-receptor PD-1 to Enhance CD8+ Cytolytic T Cell Responses
Source: Immunity. 2016 Feb 16;44(2):274–86. doi: 10.1016/j.immuni.2016.01.018 (PMC4760122; doi:10.1016/j.immuni.2016.01.018)
Supplement: Document S2. Article plus Supplemental Information [file mmc2.pdf]

Immunity

Article

Glycogen Synthase Kinase 3 Inactivation Drives T-bet-Mediated Downregulation of Co-receptor PD-1 to Enhance CD8$^+$ Cytolytic T Cell Responses

Graphical Abstract

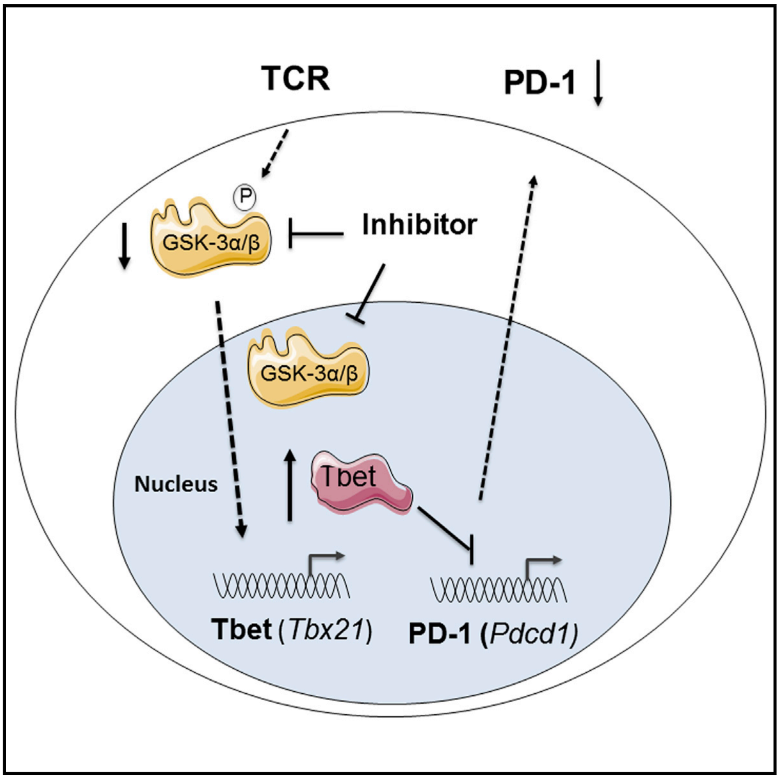

Authors

Alison Taylor, James A. Harker, Kittiphat Chanthong, Philip G. Stevenson, Elina I. Zuniga, Christopher E. Rudd

Correspondence

cer51@cam.ac.uk

In Brief

The upstream pathway regulating PD-1 expression is not clear. Rudd and colleagues show that inhibition of the serine/threonine kinase GSK-3 upregulates T-bet expression, which decreases PD-1 expression and enhances CTL function. They demonstrate that the use of GSK-3 inhibitors in vivo inhibits PD-1 and enhances T cell clearance of viral infections.

Highlights

- GSK-3 is a key upstream kinase that contributes to inhibition of PD-1 transcription
- GSK-3 siRNAs or inhibitors block PD-1 transcription to thereby enhance CTL function
- GSK-3 inhibition enhances *Tbx21* transcription, which represses PD-1 transcription
- Use of GSK-3 inhibitors in vivo downregulates PD-1 and enhances viral clearance

CrossMark
Taylor et al., 2016, Immunity *44*, 274–286
February 16, 2016 ©2016 The Authors
http://dx.doi.org/10.1016/j.immuni.2016.01.018

CellPress

Glycogen Synthase Kinase 3 Inactivation Drives T-bet-Mediated Downregulation of Co-receptor PD-1 to Enhance CD8$^+$ Cytolytic T Cell Responses

Alison Taylor,[1] James A. Harker,[2] Kittiphat Chanthong,[1] Philip G. Stevenson,[3,4] Elina I. Zuniga,[2] and Christopher E. Rudd[1,*]
[1]Cell Signalling Section, Division of Immunology, Department of Pathology, Tennis Court Road, University of Cambridge, Cambridge CB2 1QP, UK
[2]Division of Biological Sciences, University of California San Diego, La Jolla, CA 92093, USA
[3]Division of Virology, Department of Pathology, University of Cambridge, Cambridge CB2 2QQ, UK
[4]Present address: Sir Albert Sakzewski Virus Research Centre, Clinical Medical Virology Centre, School of Chemistry and Molecular Biosciences, Royal Children's Hospital and University of Queensland, Brisbane, QLD 4072, Australia
*Correspondence: cer51@cam.ac.uk
http://dx.doi.org/10.1016/j.immuni.2016.01.018
This is an open access article under the CC BY license (http://creativecommons.org/licenses/by/4.0/).

SUMMARY

Despite the importance of the co-receptor PD-1 in T cell immunity, the upstream signaling pathway that regulates PD-1 expression has not been defined. Glycogen synthase kinase 3 (GSK-3, isoforms α and β) is a serine-threonine kinase implicated in cellular processes. Here, we identified GSK-3 as a key upstream kinase that regulated PD-1 expression in CD8$^+$ T cells. GSK-3 siRNA downregulation, or inhibition by small molecules, blocked PD-1 expression, resulting in increased CD8$^+$ cytotoxic T lymphocyte (CTL) function. Mechanistically, GSK-3 inactivation increased *Tbx21* transcription, promoting enhanced T-bet expression and subsequent suppression of *Pdcd1* (encodes PD-1) transcription in CD8$^+$ CTLs. Injection of GSK-3 inhibitors in mice increased in vivo CD8$^+$ OT-I CTL function and the clearance of murine gamma-herpesvirus 68 and lymphocytic choriomeningitis clone 13 and reversed T cell exhaustion. Our findings identify GSK-3 as a regulator of PD-1 expression and demonstrate the applicability of GSK-3 inhibitors in the modulation of PD-1 in immunotherapy.

INTRODUCTION

Persistent viral infections are often associated with the functional exhaustion of virus-specific CD8$^+$ T cells (Virgin et al., 2009). Exhausted T cells have diminished effector functions and a distinct transcriptional profile relative to effector cells (Wherry, 2011). Receptor programmed death 1 (PD-1; also known as PDCD1) expression is upregulated on the surface of exhausted CD8$^+$ T cells in mice infected by the lymphocytic choriomeningitis virus clone 13 strain (LCMV-Cl13) (Barber et al., 2006; Day et al., 2006; Freeman et al., 2006; Sharpe et al., 2007). PD-1 is also upregulated during infection by the human immunodeficiency virus-1 (HIV-1) (Day et al., 2006) and hepatitis C virus (Evans et al., 2008) and in monkeys infected with the simian immunodeficiency virus (SIV) (Velu et al., 2009) and correlates with increased viral load (Barber et al., 2006; Blattman et al., 2009; Day et al., 2006; Palmer et al., 2013). Blocking antibodies against PD-1 restores CD8$^+$ T cell functionality and viral clearance (Freeman et al., 2006; Ha et al., 2008; Sharpe et al., 2007; Wherry, 2011). Checkpoint inhibitor blockade has also proven effective in the treatment of cancers such as melanoma (Hodi et al., 2003, 2010) and in combined therapy with anti-CTLA-4 (Topalian et al., 2015; Wolchok et al., 2013).

Two ligands, PD-L1 and PD-L2, have been identified for PD-1 (Freeman et al., 2000; Latchman et al., 2001; Sharpe and Freeman, 2002; Ishida et al., 2002), and PD-1 has an immunoreceptor tyrosine-based switch motif (ITSM) that binds Src homology region 2 domain-containing phosphatases SHP-1 and SHP-2 (Chemnitz et al., 2004; Okazaki et al., 2001). The preponderance of studies are compatible with a negative function for the co-receptor (Dong et al., 1999; Freeman et al., 2000; Latchman et al., 2001; Nishimura et al., 2001; Tseng et al., 2001). Co-ligation can de-phosphorylate signaling proteins (Chemnitz et al., 2004; Parry et al., 2005; Yokosuka et al., 2012) and form micro-clusters (Yokosuka et al., 2012). PD-1 can also upregulate inhibitory basic leucine zipper transcription factor, ATF-like BATF (Quigley et al., 2010), and induce motility paralysis (Zinselmeyer et al., 2013).

Despite this, the signal transduction pathway that regulates PD-1 transcription and expression in T cells has not been fully defined. Tyrosine kinases p56^{lck} and ZAP-70 activate T cells (Rudd, 1999; Weiss and Littman, 1994). Src kinase p56^{lck} binds CD4 and CD8 (Barber et al., 1989; Rudd et al., 1988; Veillette et al., 1989) and phosphorylates the TCR complex for ZAP-70 recruitment and phosphorylation of adaptors (Barber et al., 1989; Burgess et al., 1991; Chan et al., 1992; Rudd, 1999; Samelson, 2002; Weiss and Littman, 1994). By contrast, the serine/threonine kinase, glycogen synthase kinase 3 (GSK-3), first characterized in phosphorylating glycogen synthase, is constitutively active in resting T cells (Frame and Cohen, 2001; Woodgett, 1990). Two isoforms of GSK-3 (α and β) have similar kinase domains but divergent N and C termini. They influence

multiple signaling pathways although the two isoforms have distinct roles in cell survival (Frame and Cohen, 2001). In CD4$^+$ T cells, GSK-3 facilitates the exit of nuclear factor of activated T cells (NFAT) from the nucleus (Beals et al., 1997; Neal and Clipstone, 2001). TCR and CD28 phosphorylate and inactivate GSK-3 (Ohteki et al., 2000; Wood et al., 2006), and constitutively active GSK-3β (GSK-3βA9) inhibits the proliferation of T cells (Ohteki et al., 2000). GSK-3 in T cells operates independently of guanine nucleotide exchange factor VAV-1 (Wood et al., 2006).

Although certain transcription factors have been implicated in *Pdcd1* transcription, the identity of the upstream signaling event(s) that control PD-1 expression has been unclear. Here, we have identified GSK-3α and GSK-3β (hereafter referred to as GSK-3 collectively) as a key kinase that upregulated *Tbx21* transcription for the downregulation of PD-1 and enhanced CD8$^+$ cytolytic T cell function. We also demonstrated the use of small molecule inhibitors of GSK-3 to downregulate PD-1 for enhanced in vivo immunity involving the clearance of acute and chronic viral infections.

RESULTS

GSK-3 Downregulation or Inhibition Augments Cytolytic Killing of OT-I Transgenic T Cells

Although GSK-3 inhibits T cell expansion (Appleman et al., 2000; Ohteki et al., 2000; Wood et al., 2006), its role in the function of cytolytic T lymphocytes (CTLs) is not clear. To examine this, we initially examined CTL responses of T cells from OT-I transgenic mice that carry a MHC class I-restricted T cell receptor (TCR) specific for the SIINFEKL peptide of OVAlbumin ($OVA_{257\text{-}264}$) as presented by H-2k^b. T cells express the α and β isoforms of GSK-3 (Cohen and Frame, 2001). Small interfering RNAs (siRNAs) to the GSK-3α and β isoforms were used to knock down (KD) their expression in naive T cells by transfection prior to use in functional assays. siRNA treatment reduced GSK-3α and β protein substantially as seen by Western blotting (Figure 1A, iii), and by using fluorescent FITC-conjugated siRNAs, we found that more than 80% of cells had taken up the siRNAs (Figure 1A, iii). Further, siRNAs to GSK-3 increased OT-I-mediated cytolysis of mouse lymphoma cell line EL4-OVA targets significantly when compared to the control scrambled siRNA control (Figure 1A, i). There was a linear increase in killing of control samples expressing scrambled siRNAs across effector:target (E:T) ratios of 2:1 to 50:1 when assayed at day 5. This was confirmed by Scatchard plot analysis (r^2 = 0.9603) (Figure 1A, iv). By contrast, the KD of GSK-3 increased the efficiency of CTL killing. The increase in killing efficiency seen was 3- to 5-fold for E:T ratios of 2:1 and 5:1. An E:T ratio of 2:1 showed the same efficiency of killing as seen at a ratio of 25:1 for control scrambled cells (i.e., p value = 0.333). A similar potentiating effect was also evident when assayed at days 4 and 7 (data not shown). These data showed that the KD of GSK-3 markedly increased the killing capacity of CD8$^+$ OT-I cytolytic T cells.

ATP competitive and non-competitive inhibitors of GSK-3 kinase activity exist (Cohen and Goedert, 2004). SB415286 competitively inhibits both isoforms with a preference of the β isoform (Coghlan et al., 2000). Similar to the GSK-3 KD, inhibition of GSK-3 catalytic activity with SB415286 increased the killing efficiency of CTLs (Figure 1B, i). The linear increase (Figure 1B, iii) in the killing over E:T ratios was confirmed by Scatchard plot analysis (r^2 = 0.9643). Incubation of OT-I T cells with SB415286 increased maximal killing by as much as 5-fold and shifted the killing curve by 10-fold (i.e., p value = 0.111). SB415286 also increased proliferation in response to OVA peptide to the same extent as when cells were cultured with anti-PD-1, as monitored by carboxyfluorescein succinimidyl ester (CFSE) labeling (Figure S1A). Cell counting with trypan blue exclusion showed that SB415286 increased slightly the cell number in response to anti-CD3 (Figure S1B) and had no effect on cell viability (Figure S1C). These results showed that GSK-3 inactivation increases the killing efficiency of CD8-positive CTLs.

GSK-3 Downregulation or Inhibition Selectively Inhibits PD-1 Expression

We next assessed the effect of GSK-3 inhibition on receptor expression (Figure 1A, ii). GSK-3 siRNA reduced the frequency of cell surface expression PD-1 from 30% of cells to 7% of cells and also decreased the mean fluorescent intensity (MFI) of PD-1 expression without affecting CD28 or CTLA-4 expression. SB415286 reduced the frequency of cell expressing surface PD-1 expression on 54% to 7% of cells and decreased the MFI without affecting CTLA-4 or CD28 (Figure 1B, ii). Resting cells failed to express PD-1 or CTLA-4 (Figure S1D). The expression of CD44, CD62L, Tim3, CD3, BTLA, NKG2D, CD122, interleukin 2 receptor-alpha (IL-2Rα), CD25, CD69, Fas-ligand (FasL), CD8, and IL-2 was also unaffected by SB415286 (Figure 1C). Similarly, the expression of intracellular IL-2 and anti-apoptotic protein Bcl-2 was unaffected. However, consistent with enhanced CTL function, the frequency of cells expressing interferon-γ (IFN-γ), lysosomal-associated membrane protein 1 (Lamp1; CD107a), and cytotoxic T-lymphocyte-associated serine esterase 1 (Granzyme B; GZMB) was increased on OT-I CD8$^+$ T cells.

siRNA selective for the GSK-3α isoform also reduced PD-1 expression while increasing OT-I killing (Figure 1D, iii and i). It reduced GSK-3α expression as confirmed by anti-GSK-3α blotting (Figure 1D, ii) and by flow cytometry (Figure 1D, iii) without affecting GSK-3β cell surface expression. siRNA GSK-3β was also tested but failed to show specificity and was not pursued (data not shown). These data showed that GSK-3α siRNA KD alone could downregulate PD-1 and increase the killing efficiency of CTLs.

Other structurally distinct inhibitors of GSK-3 decreased PD-1 expression and potentiated OT-I killing of targets (Figure 1E). These included ATP-competitive inhibitors SB216763, CHIR99021, and L803-mts, where SB216763 has a preference for the GSK-3α isoform and CHIR99021 and L803-mts preferentially inhibited GSK-3β (Kaidanovich-Beilin and Eldar-Finkelman, 2006).

We next determined whether the reduction of PD-1 expression by GSK-3 inhibition was itself responsible for the enhanced CTL function by blocking with anti-PD-1 or PD L1-Fc (constant fragment) in the absence and presence of GSK-3 inactivation (Figure 2). PD-1 blockade increased the CTL killing efficiency to the same extent as GSK-3 siRNA as seen over multiple E:T ratios (Figure 2A). However, the addition of PD-1 or PD L1-Fc to GSK-3 siRNA-expressing cells did not increase killing beyond that seen with GSK-3 inactivation alone or vice versa. Identical results

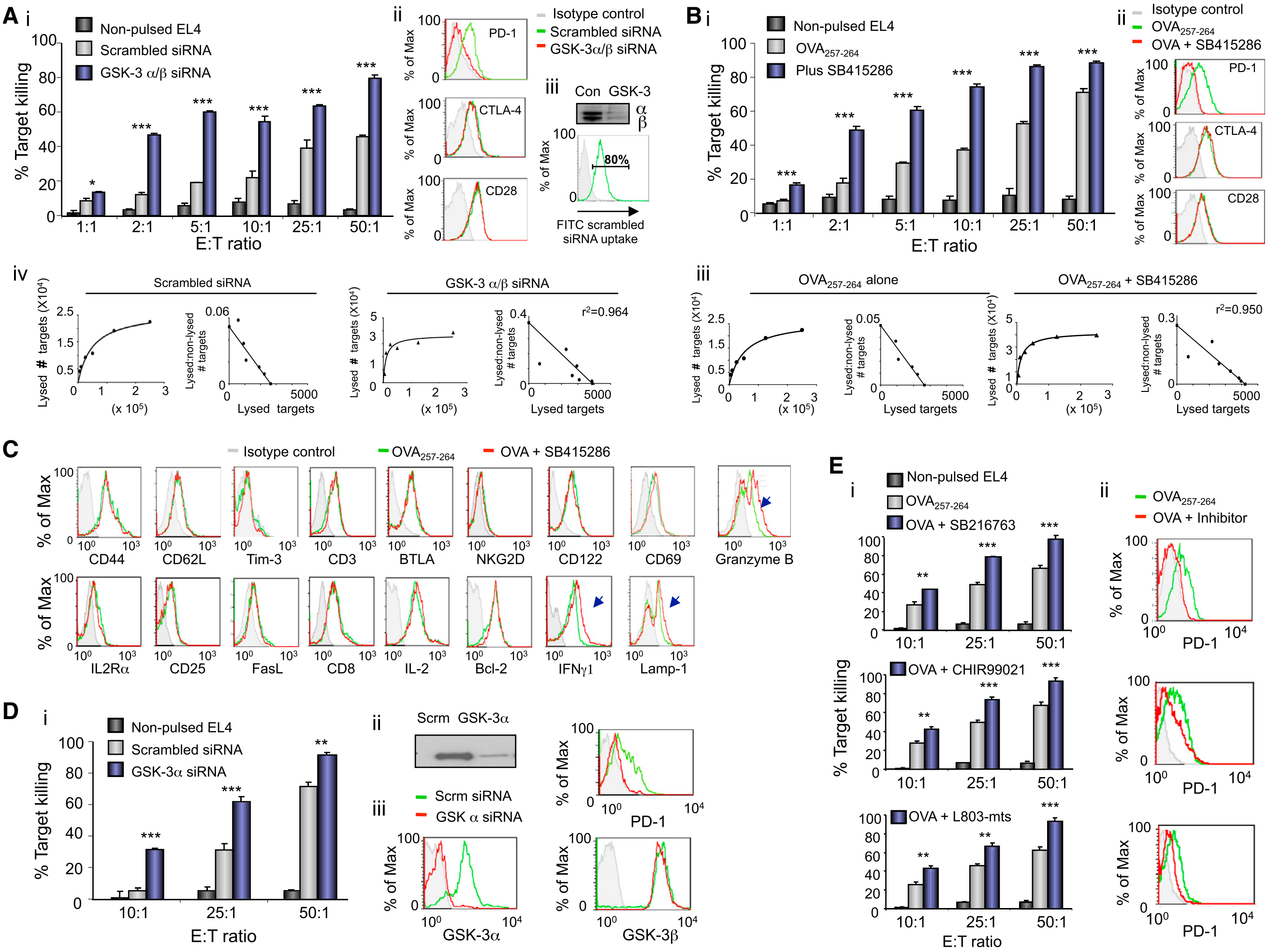

Figure 1. GSK-3 Inactivation via siRNA KD or Small Molecule Inhibitors Specifically Downregulated PD-1 Expression with Enhanced CTL Function

(A) i: siRNA against GSK3 increases the cytolytic killing of OT-I CD8$^+$ T cells. Scrambled siRNA (gray bars), siRNA against GSK3 (blue bars) (n = 5). ii: Flow cytometry profiles of receptor expression (gray line, isotype control; green line, scrambled siRNA; red line, GSK3 siRNA). iii: Anti-GSK3 blotting of cell lysates; FITC-tagged siRNA uptake. iv: Scatchard plot analysis.

(B) i: OT-I T cells were incubated without (gray bars) or with SB415286 (blue bars) (n = 5). ii: Flow cytometry profiles (as above except red line: SB415286). iii: Scatchard plot analysis.

(C) Flow cytometry profiles. Gray line, isotype control; green line, untreated cells; red line, cells incubated with SB415286.

(D) i: Cytolytic assay using GSK-3α siRNA, scrambled control siRNA (gray bars), or siRNA against GSK3α (blue bars) (n = 5). ii: GSK-3α-specific blot. iii: Flow cytometry profiles of GSK-3α, GSK-3β, and PD-1.

(E) i: Cytolytic assays using other GSK-3 inhibitors. ii: Flow cytometry profiles of PD-1 in the presence or absence of the specific inhibitor (gray line, isotype control; green line, scrambled siRNA; red line, inhibitor).

Error bars based on triplicate values in individual experiments; data shown representative of five independent experiments.

were obtained using anti-PD-1 or PDL1-Fc with SB415286 where blockade did not increase killing beyond that seen with the inhibitor, and vice versa (Figure 2B). Similar results were obtained using GSK-3 inhibitor SB216763 in combination with anti-PD-1 (Figure S2).

siRNA KD of PD-1 expression in CD8$^+$ T cells also increased cytolytic function (Figure 2D). PD-1 siRNA and SB415286 each reduced *pdcd1* transcription by >85% reduction (Figure 2C) and enhanced CTL killing to the same degree. Further, combined siRNA and SB415286 increased CTL function no further than seen with either treatment alone. These observations confirmed that the modulatory effect of GSK-3 inactivation on OT-I CTL function was primarily due to PD-1 down-modulation.

GSK-3 Downregulation or Inhibition Blocks PD-1 Transcription

We next confirmed that GSK-3 inactivation acted on *Pdcd1* transcription (encodes PD-1) in different contexts (Figure 3). Anti-CD3 ligation induces PD-1 expression on T cells (Agata et al., 1996). Two-step real-time PCR was used to separate the reverse transcription reaction from the real-time PCR assay (Wacker and Godard, 2005). The presence of SB415286 was remarkably

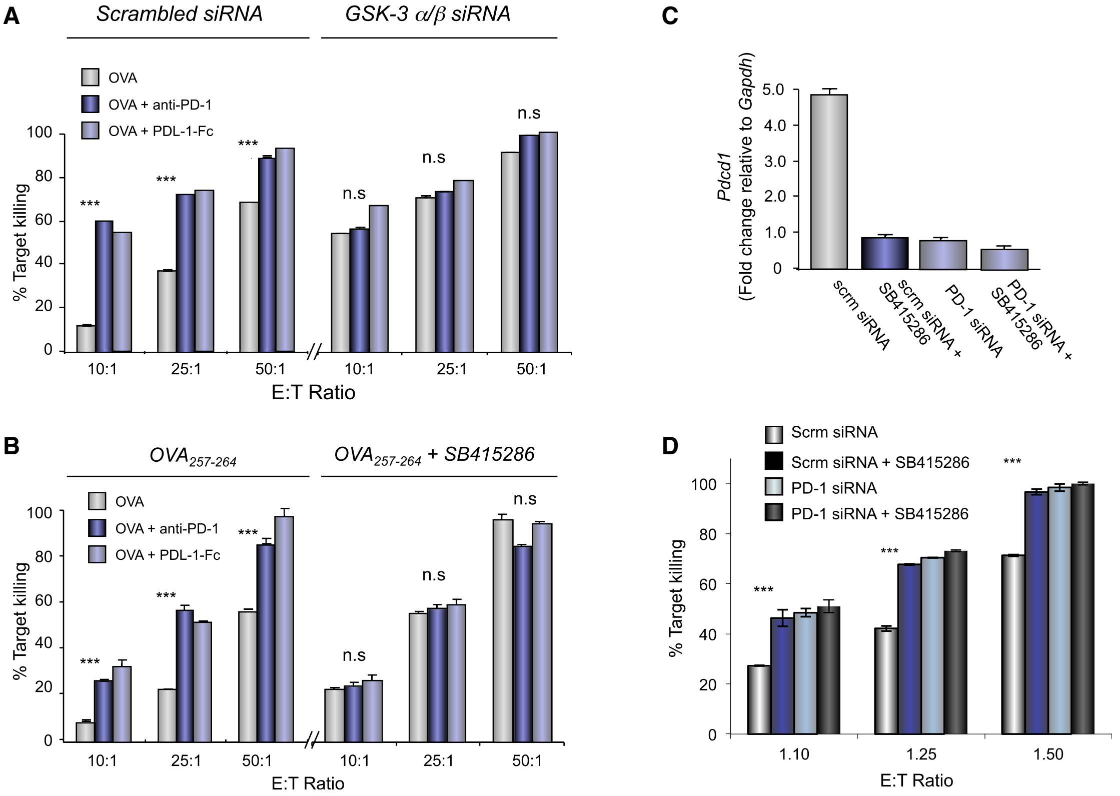

Figure 2. GSK-3 Potentiates OT-I Cytolytic Killing of EL4-OVA Target Cells via the Downregulation of PD-1

(A) Percent target killing of EL4-OVA targets by OT-I CD8$^+$ CTLs expressing scrambled or GSK-3 siRNA in the presence or absence of blocking anti-PD-1 or PDL1-Fc.

(B) Percent killing of EL4-OVA targets by OT-I CD8$^+$ CTLs incubated in the presence or absence of SB415286 with or without blocking anti-PD-1 or PDL1-Fc. OVA alone: light gray bars; anti-PD-1: dark blue bars; PD-L1-Fc: light blue bars (n = 4).

(C) Relative *Pdcd1* expression in the presence and absence of PD-1 siRNA and/or SB415286.

(D) Percent target killing from conditions in (C).

Error bars based on triplicate values in individual experiments; data shown representative of $\geq$4 independent experiments.

effective in blocking the induction of *Pdcd1* transcription by anti-CD3 over 72 hr (Figure 3A). GSK-3 siRNA also inhibited *Pdcd1* transcription in OT-I T cells in response to EL-4-OVA cells (Figure 3B). In addition, SB415286 blocked *Pdcd1* transcription in OT-I responses to EL4-OVA (Figure 3C). A titration of structurally distinct inhibitors such as CT99021, AR-AO14418, and TZD8 also inhibited *Pdcd1* transcription, whereas the phosphatase inhibitor NSC 87877 had no effect (data not shown).

Conversely, we found that SB415286 increased the transcription of *Tbx21* (encodes T-box transcription factor T-bet) concurrent with *Pdcd1* inhibition. This was observed in response to anti-CD3 ligation without affecting Tcf7-mediated transcription (Figure 3A). Similarly, GSK-3 siRNA in OT-I T cells increased *Tbx21* transcription in response to EL4-OVA (Figure 3B) as did incubation with SB415286 for 5 and 6 days (Figure 3C). Chromatin immunoprecipitation (ChIP) with two different anti-T-bet antibodies followed by PCR confirmed that GSK-3 inhibition increased T-bet binding to *Pdcd1* in anti-CD3-activated primary or Jurkat T cells (Figure 3D). Further, anti-CD3 activation of cells expressing the *Ifng CNS-12* promoter construct driven by T-bet (Kanhere et al., 2012) and incubated with SB415286 showed that increased transcription (Figure 3E). These data confirm that GSK-3 inactivation acted to increase *Tbx21* transcription and its binding to the PD-1 promoter.

We next examined whether GSK-3 mediated its effect on *Pdcd1* transcription via T-bet (Figure 3F and 3G). T-bet siRNA expression decreased the presence of T-bet transcripts while increasing PD-1 transcription consistent with its negative regulation of PD-1 (Kao et al., 2011). At the same time, SB415286 failed to reduce PD-1 expression in cells expressing T-bet siRNA, as assessed by flow cytometry. Similar findings were observed using OT-I CTL killing as a read-out (Figure 3G). T-bet siRNA impaired CTL killing over different E:T ratios as well as reducing GZMB and Lamp1 expression (Figure 3G). This inhibition was completely reversed by PD-1 blockade showing that the T-bet inhibition of CTL OT-I responses was due primarily to increased PD-1 expression. By contrast, SB415286 failed to reverse the inhibition of killing by T-bet siRNA. This result indicated that the modulatory effects of GSK-3 on PD-1 expression operated in a pathway that required the expression of T-bet.

As an additional control, we also re-expressed PD-1 in SB415286-exposed OT-I T cells and assessed whether this could reverse the enhancement of CTL killing (Figure 3H). EL-4-OVA-activated SB415286-treated cells were transfected on day 5 of

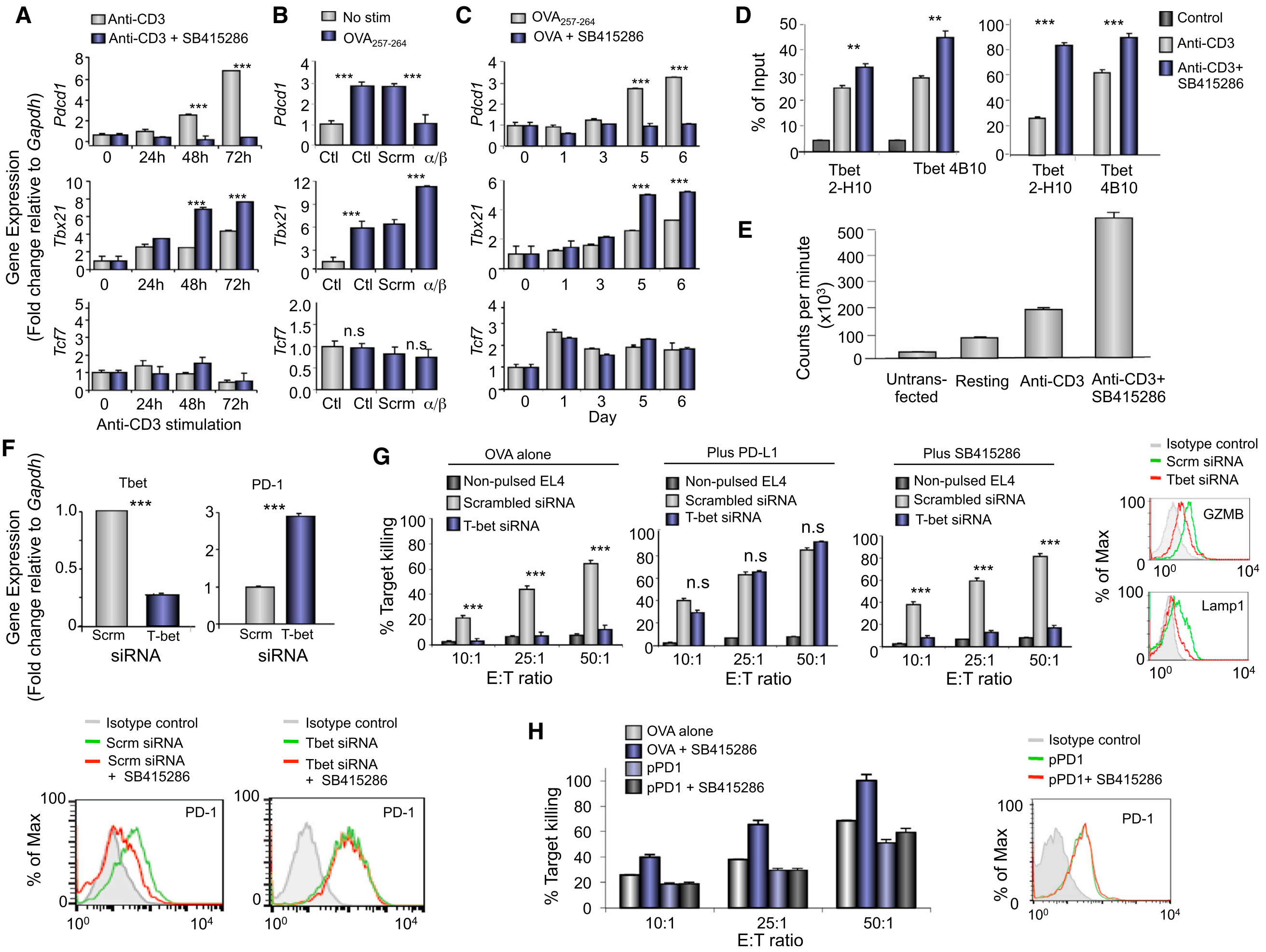

Figure 3. GSK-3 KD or Drug Inactivation Inhibits PD-1 and Increases *Tbx21* Transcription and Binding to the *Pdcd1*

(A–C) *Tbx21* and *Pdcd1* transcription in response to anti-CD3 activation with or without SB415286 (A) (n = 4), siRNA GSK-3α/β KD in OT-I cells in response to OVA (B) (n = 3), and in OT-I T cells responding to OVA peptide with and without SB415286 (C) (n = 3).

(D) ChIP using anti-T-bet antibodies 2-H10 and 4B10 followed by PCR analysis of *Pdcd1* promoter. Mouse T cells (left); Jurkat T cells (right).

(E) *Tbx21*-driven *Ifng CNS-12* promoter activity in response to CD3 with or without SB415286.

(F) Effect of T-bet siRNA on *Pdcd1* and *Tbx21* transcription (top). Flow cytometry of PD-1 expression on T cells expressing scambled siRNA or T-bet siRNA in the presence or absence of SB415286 (bottom).

(G) CTL killing efficiency of T-bet siRNA-expressing CTLs in the presence of anti-PD-L1 blockade and/or SB415286 and on expression of GZMB and Lamp1 expression in T-bet siRNA-expressing OT-I cells (n = 3).

(H) Killing efficiency of OT-I CTLs exposed to SB415286 followed by pPD-1 expression and flow cytometric profile of restored PD-1 expression (n = 3).

Error bars based on triplicate values in individual experiments; data shown representative of ≥3 independent experiments.

culture with PD-1 followed by an assessment of cytolysis 48 hr later. The SR alpha promoter plasmid with PD-1 (pPD1) induced PD-1 expression similar to levels on activated T cells. This expression reversed the enhanced killing induced by SB415286, confirming that PD-1 downregulation was responsible for the potentiating effects of GSK-3 inactivation on CTL function.

Another possible connection to GSK-3 was the transcription factor NFATc1 because GSK-3 inhibition increases nuclear NFATc1 in CD4$^+$ T cells (Beals et al., 1997; Neal and Clipstone, 2001). *Pdcd1* expression is regulated by NFAT2 (Oestreich et al., 2008) and NFAT binding sites exist in*Tbx21* (Martinez et al., 2015). NFATc1 siRNA downregulated *nfat* transcripts (Figure S3A). However, unlike GSK-3 inhibition, NFATc1 siRNA inhibited all events including T-bet (Figure S3B) and PD-1 (Figure S3C) transcription and CTL killing (Figure S3D). Cyclosporin A (CsA) treatment also inhibited *Tbx21*, *Pdcd1*, and CTL killing and SB415286 was unable to rescue the inhibitory effect (Figures S4A–S4D).

In Vivo GSK-3 Inhibition Decreases PD-1 Expression and Enhances CTL Function of CD8$^+$ OT-I T Cells

To assess the in vivo effect of GSK-3 inactivation on PD-1 expression, we initially examined the in vivo generation of OT-I responses to OVA peptide in the presence of SB415286. OVA peptide was injected intravenously (i.v.) into OT-I transgenic mice with and without SB415286 followed by the harvest of

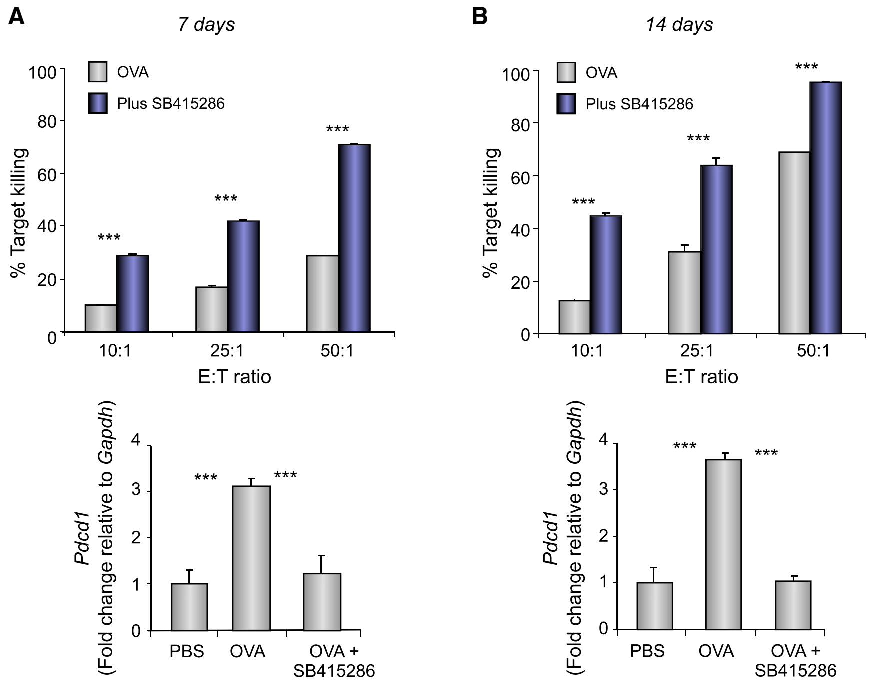

Figure 4. GSK-3 Inactivation In Vivo Suppresses PD-1 and Increases T-bet Expression Concurrent with Enhanced OT-I CTL Function

SB415286 administered in vivo on days 0 or 7 and cells purified ex vivo on days 7 or 14. Ex vivo purified T cells were then assessed for cytolytic activity (percent target killing) and qPCR for *Pdcd1* expression on days 7 (A) and 14 (B). Mean and SD of six mice per group.

spleens and lymph nodes (LNs) at day 7 (Figure 4A) or a repeat injection of the drug at day 7 followed by a harvest on day 14 (Figure 4B). T cells from extracted spleens/LNs were then assessed for the ex vivo killing of EL4-OVA targets. From this, the in vivo injection with SB415286 resulted in a 2- to 5-fold increased killing efficiency of ex vivo extracted CTLs over a range of E:T ratios. qPCR of extracted T cells also confirmed that in vivo exposure to SB415286 inhibited *Pdcd1* transcription. These data provided initial evidence that the in vivo inhibition of GSK-3 with a small molecular inhibitor suppressed PD-1 expression for enhanced CTL function.

GSK-3 Regulates Clearance of Herpes MHV-68 Virus Infection via PD-1 Downregulation

We were next interested in whether small molecule inhibitors of GSK-3 could treat viral infections via downregulation of PD-1. To test this, we infected BALB/c mice intra-nasally with the luciferase$^+$ Murid herpesvirus 68 (MHV-68), an isolate of Murid herpesvirus 4 (MuHV-4) (Stevenson et al., 1999), in the presence or absence of SB415286. Viral luciferase expression effectively tracks the spread of infection as monitored by luciferin injection and CCD camera scanning (Milho et al., 2009). The presence of virus was followed over time from the nose to lung alveoli where lytic replication occurs. In the MHV-68 model, day 7 corresponds to the latent period and day 14 to chronic infection (Stevenson et al., 1999). We found that SB415286 markedly reduced the luciferase signal in the nose and lungs in mice as assessed on day 7 (Figures 5A and S5B). Quantitative comparisons of maximum radiance confirmed an increase in the presence of luciferase$^+$ MHV-68 in lungs from day 3 to 7, which drug treatment reduced from $10^{6\text{-}7}$ to $10^{4\text{-}5\ ps\text{-}1}$. The viral load (plaque forming units [pfu]) was also significantly reduced from 6×10^4 to 1×10^4 at day 7 and from 2×10^4 to 4×10^3 at days 7 and 14 (Figure 5B). Quantitative real-time PCR of T cells extracted from spleens of SB415286-treated mice showed a reduction in *pdcd1* transcription concurrent with an increase in T-bet expression (Figure 5C). Moreover, splenic T cells from mice treated with SB415286 and tested ex vivo for the killing of targets pulsed with MHV-68-derived peptide $M2_{91\text{–}99}$ showed an increase in killing capacity (Figure 5D).

To assess whether the GSK-3 modulation of PD-1 expression was responsible for this effect, we next conducted the same experiment using a combination of anti-PD-1 blockade with SB415286 (Figure 5E). Anti-PD-1 blockade reduced viral spread as seen by the reduction in max radiance (i.e., from 4×10^7 to $8 \times 10^{5\ ps\text{-}1}$) when assessed on days 3, 5, and 7. SB415286 reduced the maximum radiance to the same degree, and importantly, when used in combination, anti-PD-1 blockade had no further effect than SB415286, and vice versa. Measurement of viral titers at days 7 and 14 showed that anti-PD-1 blockade and SB415286 reduced viral titers to the same extent (Figure 5F). Further, the injection of anti-PD-1 did not increase the response further in mice injected with SB415286 and vice versa. These observations showed that the effect of GSK-3 inhibition in vivo in response to MHV-68 was mediated via PD-1 downregulation.

Lastly, the increase in viral clearance due to GSK-3 inhibition was accompanied by an increase in Lamp1 (i.e., from 120 to 210 GeoMFI, $p < 0.001$), GZMB (i.e., from 11 to 14GeoMFI, $p < 0.001$), and IFN-γ (i.e., from 29 to 43 GeoMFI, $p < 0.05$) expression (Figure 5G). By contrast, the presence of SB415286 did not alter the frequency of cells expressing CD44 and CD62L (Figure 5H), in accord with studies on *Pdcd1*$^{-/-}$ T cells (Keir et al., 2007). Cells expressing CD4 also did not change. By contrast, $M2_{91\text{–}99}$ tetramer staining showed increased numbers of M2 peptide-specific CD8$^+$ T cells (Figure 5I). Overall, our findings show that the in vivo inactivation of GSK-3 can significantly reduce the progression of MHV-68 infection in mice due to reduced PD-1 expression.

GSK-3 Augments Clearance of Chronic LCMV-Cl13 Infection

Exhausted virus-specific CD8$^+$ T cells during chronic infection are characterized by prolonged expression of PD-1 in response to the lymphocytic choriomeningitis virus variant LCMV-Cl13 (Ahmed et al., 1984; Barber et al., 2006; Day et al., 2006). This contrasted with the LCMV Armstrong strain (LCMV-Arm) that

CellPress

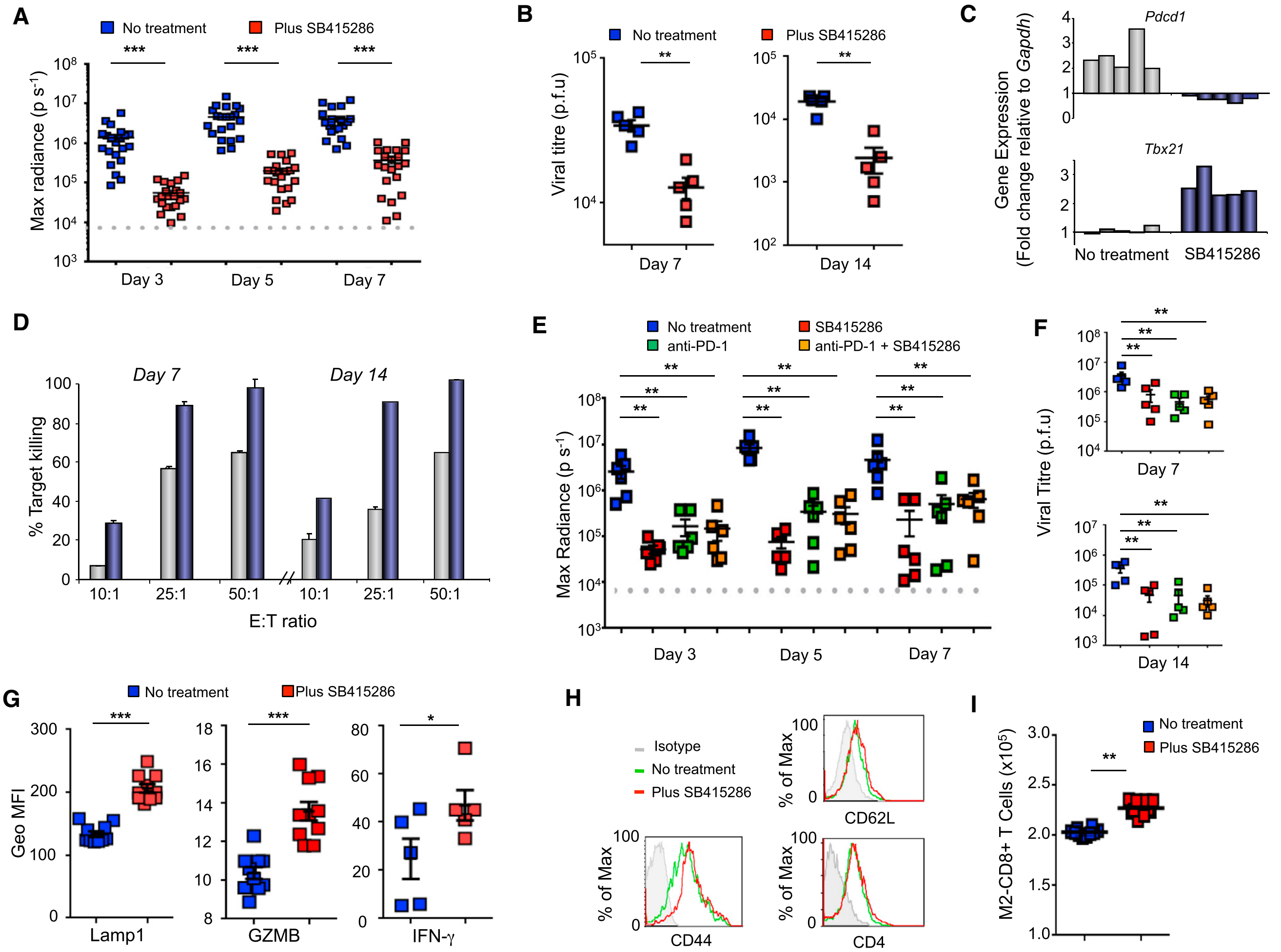

Figure 5. In Vivo GSK-3 Inhibition Increased Acute MHV-68 Viral Clearance via PD-1 Downregulation

BALB/c mice were intra-nasally infected with MHV-68 with/without an i.p. injection of SB415286.

(A) Histogram showing maximal radiance values on days 3, 5, and 7 (n = 20).

(B) Histogram showing viral titers at days 7 and 14 (n = 5).

(C) qPCR values of *Pdcd1* and *Tbx21*. Gray bars, non-treated; blue bars, SB415286 treated. Each column represents a different mouse.

(D) CTL killing of EL4-M2 cells (percent target killing). T cells isolated from spleen were assessed for ex vivo killing of EL4-M2 cells (n = 3).

(E and F) BALB/c mice were infected with MHV-68 and treated with anti-PD1 with and without SB415286 undergoing the same regime as in (A) (n = 3). Radiance values on days 3, 5, and 7 (E); viral titers on days 7 and 14 (F).

(G) Flow cytometry profile of Lamp1, GZMB, and IFN-γ expression (n = 3).

(H) Flow cytometry profiles of CD44, CD62L, and CD4 expression. Gray line, isotype control; green line, T cells stimulated with OVA peptide alone; red line, T cells stimulated with OVA peptide in the presence of SB415286.

(I) Absolute numbers of M2 peptide-specific $CD8^+$ T cells.

Mean and SD of at least five mice per group. Also see Figures S5A and S5B.

induces a more robust response and resolves within 8–10 days. To assess the role of the GSK-3-T-bet-PD-1 pathway in LCMV-Cl13 infection, mice were infected i.v. with the LCMV-Cl13 variant followed by an injection with SB415286 at day 25 when exhaustion had been established in the chronic phase of the infection (Figure S5C). Mice were then monitored on days 23, 25, 30, and 37. $DbGP_{33\text{-}41}$ peptide staining of $CD8^+$ T cells from mice infected with LCMV-Cl13 demonstrated high expression of PD-1, unlike T cells from LCMV-Arm-infected mice. Further, $D^bGP_{33\text{-}41}{}^+CD8^+$ T cells from mice treated with SB415286 showed a significant reduction in global PD-1 expression relative to untreated mice, when assayed on days 25 and 30 (Figure 6A). PD-1 expression was inversely related with an increase in T-bet expression ($r^2 = 0.7$) (Figure 6B). Consistent with PD-1 downregulation, SB415286-treated mice had a significant reduction in viral load in blood at days 30 and 37 (Figure 6C). When expressed as percent infectivity compared to day 23 (Figure 6D), mice showed a range of responses with more than a log reduction between some untreated and treated mice (i.e., 10^2 from control mice to 5×10^{-2}), as reported in response to anti-PD-1 (Brooks et al., 2008; Ha et al., 2008; West et al., 2013). This was accompanied by a slight increase in the number of $GP_{33\text{-}41}{}^+CD8^+$ T cells

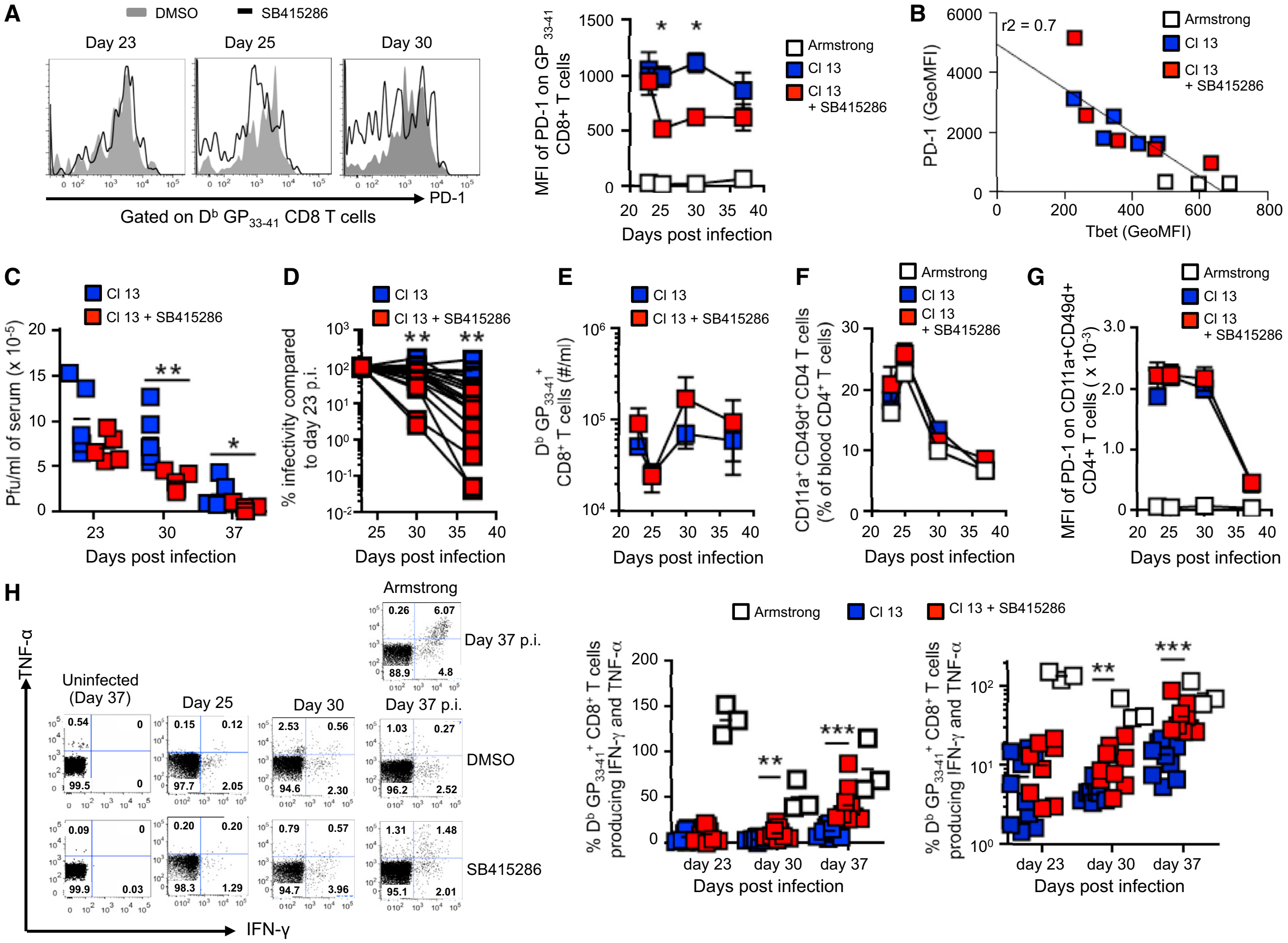

Figure 6. GSK-3 Inhibition In Vivo Can Enhance Viral Clearance in Chronic Infection by LCMV-Cl13

Mice were infected i.v. with LCMV-Cl13 and treated with/without SB415286.

(A) Flow cytometry depicting PD-1 expression on D^bGP_{33-41} $CD8^+$ T cells in LCMV-Cl13-infected mice treated with and without SB415286. Right plot represents MFI values for PD-1 expression D^bGP_{33-41} $CD8^+$ T cells at 23–40 days after infection (n = 2). Blue box, DMSO-treated LCMV-Cl13-infected mice; red box, SB415286-treated LCMV-Cl13-infected mice; white box, LCMV-Arm control.

(B) Inverse relationship between PD-1 and T-bet in mice treated with SB415286 (boxes as in A; each box represents a different mouse).

(C) Viral load in serum of LCMV-Cl13-infected mice treated with or without SB415286. pfu/ml viral titers in serum ($\times 10^5$) days 23, 30, and 37.

(D) Percent infectivity at days 30 and 37 compared to day 23.

(E) Number of D^bGP_{33-41} CD8 T cells taken from the spleens of mice, treated with or without SB415286 over 30–40 days.

(F and G) Number of $CD4^+$ T cells ($CD11a^+CD49d^+CD4^+$) (F) and total number of $CD4^+$ cells (G) from mice infected with LCMV-Cl13 and treated with or without SB415286.

(H) Flow cytometry profiles of IFN-γ- and TNF-α-expressing T cells from mice infected with LCMV-Arm control (day 37) or LCMV-Cl13 (day 25, 30, 37). Histograms (linear scale and log scale) showing the numbers of D^bGP_{33-41} $CD8^+$ T cells expressing IFN-γ and TNF-α after GP_{33-41} peptide stimulation of PBMCs ex vivo from untreated and SB415286-treated mice. Blue boxes, DMSO-treated mice; red boxes, SB415286 treated; white boxes, LCMV-Arm control. Each box represents a different mouse. Data represent two combined independent experiments of n $\geq$ 4 mice per group.

Statistics for (B) were done by 2-way ANOVA, (C) and (E) by non-parametric Mann-Whitney at each time point. *$p < 0.05$, **$p < 0.01$, ***$p < 0.001$.

(Figure 6E). By contrast, SB415286 treatment had no obvious effect on cell number (Figure 6F) or PD-1 expression (Figure 6G) of $CD4^+$ T cells ($CD11a^+CD49d^+CD4^+$) from mice infected with LCMV-Cl13. LCMV-specific $CD4^+$ T cells express $CD11a^{hi}CD49d^+$ (McDermott and Varga, 2011).

Further, $GP_{33-41}{}^+CD8^+$ T cells showed a significant increase in the percentage of cells expressing IFN-γ and tumor necrosis factor-alpha (TNF-α) at days 30 and 37 induced by SB415286 (Figure 6H). As a positive control, the Armstrong strain of the virus also showed increased numbers of cells expressing IFN-γ and TNF-α. These data showed that the inhibition of the GSK-3 pathway can also modulate PD-1 expression during LCMV-Cl13 chronic infection that is associated with T cell exhaustion.

DISCUSSION

Despite the importance of PD-1 in modulating T cell responses, the upstream pathway responsible for PD-1 downregulation

expression has yet to be defined. Here, we have identified GSK-3 as a key upstream kinase that regulated PD-1 expression and enhanced CD8$^+$ CTL function for the in vivo clearance of MHV-68 and LCMV-Cl13 viral infections. GSK-3 inactivation operated in a pathway that increased *Tbx21* transcription that in turn inhibited *Pdcd1* transcription and expression for enhanced CD8$^+$ CTL killing efficiency. We also demonstrated the use of small molecular inhibitors of GSK-3 to reduce PD-1 expression for enhanced in vivo immunity.

Previous studies have shown that GSK-3β inhibits the proliferation and development of CD4$^+$ T cells (Beurel et al., 2011; Ohteki et al., 2000; Schroeder et al., 2013; Wood et al., 2006). Our findings provided an additional mechanism to account for inhibition where inactivation of GSK-3α/β downregulates PD-1, thereby removing the inhibitory effect of the co-receptor on proliferation. Its downregulation by siRNA, or chemical inhibition, potently inhibited PD-1 transcription in CD8$^+$ T cells resulting in an enhancement of OT-I killing of targets by 5- to 10-fold. In a screen of receptors, only the expression of PD-1 on CD8$^+$ T cells was inhibited. PD-1 blockade, either in vitro or in vivo, did not increase the enhanced killing efficiency of CTLs induced by GSK-3 inactivation, or vice versa. If GSK-3 operated primarily via a different pathway, additional effects of GSK-3 inhibition beyond PD-1 blockade would have been expected. Further, the ectopic PD-1 expression completely reversed the enhancement of killing efficiency induced by GSK-3 inhibition. Although GSK-3 can regulate the expression of numerous other genes, our findings underscored the dominance of GSK-3's effect on PD-1 on CD8$^+$ CTL function. In accord, PD-1 has previously been shown to inhibit the generation of cytolytic T cells (Okazaki et al., 2002). A range of structurally distinct competitive and non-competitive inhibitors inhibited PD-1 transcription, whereas the phosphatase inhibitor NSC 87877 had no effect (data not shown).

Our findings also demonstrated the in vivo application of small molecule inhibitors of GSK-3 for the downregulation of PD-1 in immune therapy. Injection of SB415286 and other inhibitors enhanced OT-I CTL in vivo responses, as well as the clearance of acute or chronic infection by MHV-68 and LCMV-Cl13. GSK-3 inhibition reduced PD-1 expression and LCMV-Cl13 viral titer by a half log when administered during the exhaustion phase of infection. As with anti-PD-1 blockade, a range of effect was seen among individual mice with as much as a log reduction in infection between control and SB415286-treated mice. These effects were similar to those reported with VV/GP33 (Ha et al., 2008) and in anti-PD1-treated mice (i.e., a half log difference) (Brooks et al., 2008) as well as in *Tbx21*$^{-/-}$ mice (Kao et al., 2011). GSK-3 inhibition also restored the functionality of exhausted $GP_{33\text{-}41}$$^+$CD8$^+$ T cells as evidenced by the increased expression of TNF-α and IFN-γ. The connection to IFN-γ is intriguing given reports of an impairment of IFN-γ in patients carrying certain PD-1 polymorphisms (Kroner et al., 2005). Our findings suggest that GSK-3 inhibitors could be used to substitute or complement anti-PD-1 or PD-L1 therapy presently in use in infection and cancer (Barber et al., 2006; Day et al., 2006; Freeman et al., 2006; Grakoui et al., 2006; Ha et al., 2008; Wolchok et al., 2013) or in combination with CTLA-4 blockade (Topalian et al., 2015; Wolchok et al., 2013).

Mechanistically, we found that GSK-3 inhibition operated by increasing the transcription of T-bet, a central regulator of Th1 cell differentiation (Glimcher, 2007). Surprisingly, this connection between GSK-3 and T-bet had not previously been reported and could have a range of functional implications to T cell differentiation and function. In CD8$^+$ T cells, GSK-3 inactivation increased Tbx21 transcription for enhanced T-bet expression that in turn inhibited *Pdcd1* transcription. We showed by ChIP analysis that GSK-3 inactivation increased T-bet binding to the *Pdcd1* promoter and confirmed with siRNAs that T-bet negatively regulates *Pdcd1* transcription (Kao et al., 2011; Wherry et al., 2007). Importantly, we found that GSK-3 downregulation of PD-1 was dependent upon T-bet expression as shown by the inability of SB415286 to downregulate PD-1 on T-bet siRNA-expressing cells. In our model, GSK-3 acts as a suppressor of a suppressor of a suppressor, namely PD-1. Upon activation, GSK-3 is inhibited by TCR/CD28-induced phosphorylation leading to increased T-bet expression and the development of effector function. In the case of CD28, GSK-3 inactivation involves its binding and activation of phosphatidyl-inositol 3 kinase (PI3K) and protein kinase B (AKT) (Prasad et al., 1994; Rudd and Schneider, 2003). However, the inactivation process is inefficient, allowing the induction of some PD-1 expression via the general activation of T cells. The application of drug or interfering RNAs allowed for a more complete inactivation of GSK-3 and reduced PD-1 expression. Remarkably, we also noted that PD-1 blockade reversed T-bet siRNA inhibition of OT-I CD8$^+$ CTL responses, indicating that altered PD-1 expression can account for much of the T-bet regulation of CTL function.

It also appeared that T-bet did not affect the transcription of all its potential promoters in CD8$^+$ T cells. For example, although T-bet binds to the Tim3 promoter (Anderson et al., 2010), we saw no consistent effect of GSK-3 inhibition on Tim3 expression. This could be related to chromosomal accessibility or other factors affected by GSK-3 signaling. However, despite the dominance of PD-1 in CD8$^+$ T cells, GSK-3 could potentially still directly affect other genes that might alter CTL function in other conditions such as cancer. For example, T-bet binding sites exist in the GZMB and IFN-γ promoters (Martinez et al., 2015). In other words, an increase in GZMB and IFN-γ is due to PD-1 downregulation with an additional contribution direct GSK-3 upregulation of GZMB and IFN-γ gene expression. We did not observe an effect on CD44 or CD62L expression, agreeing with reports of normal CD44/CD62L expression on *pdcd1*$^{-/-}$ T cells (Keir et al., 2007).

The connection of GSK-3-T-bet-PD-1 axis to other signaling events is also possible. The transcription factor FoxO1 sustains expression of PD-1 during chronic LCMV-Cl13 infection involving increased PKB/AKT activity (Staron et al., 2014). Notch positively regulates PD-1 transcription (Mathieu et al., 2013), whereas activator protein 1 (AP1) and Blimp1 (B-lymphocyte maturation protein 1) suppresses it (Lu et al., 2014; Xiao et al., 2012). By contrast, although cytokine receptors increase PD-1 expression via STATs (Signal transducers and activators of transcription) (Kinter et al., 2008), these do not obviously intersect with antigen-receptor signaling in T cells.

In CD4$^+$ T cells, GSK-3 inhibition increases the residency of nuclear NFATc1 (Beals et al., 1997; Neal and Clipstone, 2001), whereas *Pdcd1* expression is regulated by NFAT2 (Oestreich

et al., 2008). Whether this pathway is connected to GSK-3 regulation of PD-1 in effector CD8+ T cells is unclear because we failed to observe an effect of GSK-3 inactivation on PD-1 expression on CD4+ T cells during LCMV-Cl13 infection. Whether this lack of an effect will be seen in other in vivo contexts needs to be clarified. The situation is also complicated by the fact that NFATc1 regulates many events (Macián et al., 2001; Masuda et al., 1998). In our hands, NFATc1 inactivation inhibited T-bet, PD-1 transcription, and CTL killing; this contrasts with GSK-3 inactivation in CD8+ T cells, where PD-1 transcription was inhibited but T-bet transcription and CTL function were increased. An NFAT1 mutant unable to bind to AP-1 promotes CD8+ T cell exhaustion (Martinez et al., 2015). NFAT2 has been reported to be primarily nuclear in tolerant CD8+ T cells (Srinivasan and Frauwirth, 2007), although others have reported that NFAT2 is not nuclear in exhausted T cells (Agnellini et al., 2007). We previously found a connection between NFATc1 and adaptors ADAP and SKAP1 in PD-1 expression indirectly due to suboptimal conjugation and activation of T cells (Li et al., 2015). These different findings underscore a complexity of NFAT in interfacing with different activation events in T cells. Overall, the full therapeutic potential of GSK-3 inhibitors in the downregulation of PD-1 in the treatment of infection and cancer remains to be clarified.

EXPERIMENTAL PROCEDURES

Mice

C57BL/6 and BALB/c (Harlan UK) mice were housed at the Central Biological Services (Cambridge University). Mice were infected when 6–8 weeks old under the Home Office Project License 80/2189. Antibodies and GSK-3 inhibitors are described in the Supplemental Experimental Procedures.

In Vitro Assays

Primary mouse T cells (OT-I, C57/b6) were isolated from spleens and cultured in vitro in supplemented RPMI 1640 (Raab et al., 2010; Veale et al., 1999). T cell enrichment was performed with T cell purification columns (R&D Systems). OVA-specific CD8+ cytolytic T cells were generated by incubating OT-I splenocytes with SIINFEKL peptide of OVA ($OVA_{257-264}$) at 10 ng/mL for 5–7 days, usually in experiments using SB415286 such as in Figure 1B. Alternatively, EL-4 cells were incubated with 10 nM $OVA_{257-264}$ peptide (Bachem) for 1 hr at 37°C and treated with mytomycin C (Sigma-Aldrich) (final concentration of 10 μg/mL) prior to mixing with primary T cells by co-culturing at a ratio of 1:5 of EL4 and T cells in order to generate cytotoxic T cells.

CTLs were generated in the presence or absence of inhibitors and/or anti-PD-1 or anti-PDL-1 blockade for 5–7 days prior to washing and analysis by FACs or PCR or in cytoxicity assays. In some cases, naive OT-I T cells were first incubated with OVA peptide with or without GSK-3 inhibitors and/or anti-PD-1 or PD-L1-Fc (1–3 μg/mL). 25 mM stock solutions were prepared in DMSO and diluted to a concentration of 1–10 μM in vitro. NSC 87877 was used at 50 μM (*Tocris* Bioscience *Boston* Biochem) (Song et al., 2009) and cyclosporin A (CsA) (Sigma) at 0.5 μg/mL.

In certain cases, naive cells were subjected to nuclear transfection in the presence of various siRNA oligos (i.e., GSK-3). 3.0–5.0 μg of siRNAs were added to 1 × 10^6 T cells and suspended in 100 μL of nucleofector solution for T cells (Amaxa Biosystems). Cells were electroporated with a Nucleofector (Amaxa Biosystems), as previously described (Smith et al., 2013; Valk et al., 2006). In certain instances, pre-activated T cells were transfected with mouse PD-1 (Takebe et al., 1988).

For in vitro cytotoxic assays, transfected T cells were plated in 96-well plates at the start of culture with activating EL4 cells pulsed with OVA peptide except in the case of the MHV-68 in which EL4s were pulsed with the M2 peptide ($M2_{91-99}$, GFNKLRSTL) (SIGMA-Genosys) (Husain et al., 1999). Cytotoxicity was assayed with a Cytotox 96 nonradioactive kit (Promega) according to the instructions provided.

Luciferase reporter assays were performed with Dual-luciferase Reporter Assay System (Promega).

Quantitative Real-Time PCR and Chromatin Immunoprecipitation

Single-strand cDNA was synthesized with an RT-PCR kit (QIAGEN) and phases of PCR amplification were monitored to ensure a measurement of real-time transcription (Wacker and Godard, 2005). ChIP (Pierce Agarose ChIP kit) was conducted according to the manufacturer's protocols (Thermo Scientific #26156).

Viruses and In Vivo Luciferase Imaging

Luciferase tagged-MHV-68 viral stock isolation, infection, and imaging were conducted as described (de Lima et al., 2004) and in Supplemental Experimental Procedures. Mice received treatment on days 0, 3, 5, 7, and 10 of either PBS, SB415286 (10 μg/kg), or anti-PD1/PD-L1 (100 μg per dose/mouse) intraperitoneally (i.p.). For luciferase imaging, mice were injected i.p. with luciferin (2 μg per mouse), anesthetized with isoflurane, and scanned with an IVIS Lumina (Caliper Life Sciences) as previously described (Milho et al., 2009). For LCMV, LCMV-Arm and LCMV-Cl13 strains were prepared as described (Harker et al., 2011, 2013). Mice were infected i.v. with 2 × 10^6 pfu of LCMV-Arm or LCMV-Cl13. Mice received doses of SB415286 or PBS every 48 hr from day 23 until day 37. LCMV titers in the serum were determined by vero cell plaque assays as described previously. Viruses were also grown, identified, and quantified as described (Ahmed et al., 1984; Borrow et al., 1995).

In Vivo Priming OT-I Tg Cells

OVA peptide (1 μg) was injected i.v. into OT-I Tg mice with and without SB415286 (10 μg) on days 0 and 7. T cells were purified from spleens harvested on days 7 and 14.

SUPPLEMENTAL INFORMATION

Supplemental Information includes five figures and Supplemental Experimental Procedures and can be found with this article online at http://dx.doi.org/10.1016/j.immuni.2016.01.018.

AUTHOR CONTRIBUTIONS

A.T. and C.E.R. conceived and designed experiments. A.T. conducted the majority of in vitro experiments and C.E.R. wrote the majority of the manuscript. A.T., P.G.S., and C.E.R. designed and A.T. conducted experiments on MHV-68, and J.A.H., A.T., and E.I.Z. designed and conducted LCMV-Cl13 experiments and contributed to writing the relevant section. K.C. conducted experiments on NFATc1 with A.T. and C.E.R.

ACKNOWLEDGMENTS

C.E.R. was supported by Wellcome Trust 092627/Z/10/Z, J.A.H. by an Irvington Institute Postdoctoral Fellowship from the Cancer Research Institute (New York), and E.I.Z. by a Leukemia and Lymphoma Society Scholar Award and a grant from the NIH AI081923. We thank Dr. Graham Lord (King's College London) for the kind gift of the *Ifng CNS-12* promoter.

Received: July 20, 2014
Revised: May 12, 2015
Accepted: November 11, 2015
Published: February 16, 2016

REFERENCES

Agata, Y., Kawasaki, A., Nishimura, H., Ishida, Y., Tsubata, T., Yagita, H., and Honjo, T. (1996). Expression of the PD-1 antigen on the surface of stimulated mouse T and B lymphocytes. Int. Immunol. *8*, 765–772.

Agnellini, P., Wolint, P., Rehr, M., Cahenzli, J., Karrer, U., and Oxenius, A. (2007). Impaired NFAT nuclear translocation results in split exhaustion of virus-specific CD8+ T cell functions during chronic viral infection. Proc. Natl. Acad. Sci. USA *104*, 4565–4570.

Ahmed, R., Salmi, A., Butler, L.D., Chiller, J.M., and Oldstone, M.B. (1984). Selection of genetic variants of lymphocytic choriomeningitis virus in spleens of persistently infected mice. Role in suppression of cytotoxic T lymphocyte response and viral persistence. J. Exp. Med. *160*, 521–540.

Anderson, A.C., Lord, G.M., Dardalhon, V., Lee, D.H., Sabatos-Peyton, C.A., Glimcher, L.H., and Kuchroo, V.K. (2010). T-bet, a Th1 transcription factor regulates the expression of Tim-3. Eur. J. Immunol. *40*, 859–866.

Appleman, L.J., Berezovskaya, A., Grass, I., and Boussiotis, V.A. (2000). CD28 costimulation mediates T cell expansion via IL-2-independent and IL-2-dependent regulation of cell cycle progression. J. Immunol. *164*, 144–151.

Barber, E.K., Dasgupta, J.D., Schlossman, S.F., Trevillyan, J.M., and Rudd, C.E. (1989). The CD4 and CD8 antigens are coupled to a protein-tyrosine kinase (p56lck) that phosphorylates the CD3 complex. Proc. Natl. Acad. Sci. USA *86*, 3277–3281.

Barber, D.L., Wherry, E.J., Masopust, D., Zhu, B., Allison, J.P., Sharpe, A.H., Freeman, G.J., and Ahmed, R. (2006). Restoring function in exhausted CD8 T cells during chronic viral infection. Nature *439*, 682–687.

Beals, C.R., Sheridan, C.M., Turck, C.W., Gardner, P., and Crabtree, G.R. (1997). Nuclear export of NF-ATc enhanced by glycogen synthase kinase-3. Science *275*, 1930–1934.

Beurel, E., Yeh, W.I., Michalek, S.M., Harrington, L.E., and Jope, R.S. (2011). Glycogen synthase kinase-3 is an early determinant in the differentiation of pathogenic Th17 cells. J. Immunol. *186*, 1391–1398.

Blattman, J.N., Wherry, E.J., Ha, S.J., van der Most, R.G., and Ahmed, R. (2009). Impact of epitope escape on PD-1 expression and CD8 T-cell exhaustion during chronic infection. J. Virol. *83*, 4386–4394.

Borrow, P., Evans, C.F., and Oldstone, M.B. (1995). Virus-induced immunosuppression: immune system-mediated destruction of virus-infected dendritic cells results in generalized immune suppression. J. Virol. *69*, 1059–1070.

Brooks, D.G., Ha, S.J., Elsaesser, H., Sharpe, A.H., Freeman, G.J., and Oldstone, M.B. (2008). IL-10 and PD-L1 operate through distinct pathways to suppress T-cell activity during persistent viral infection. Proc. Natl. Acad. Sci. USA *105*, 20428–20433.

Burgess, K.E., Odysseos, A.D., Zalvan, C., Druker, B.J., Anderson, P., Schlossman, S.F., and Rudd, C.E. (1991). Biochemical identification of a direct physical interaction between the CD4:p56lck and Ti(TcR)/CD3 complexes. Eur. J. Immunol. *21*, 1663–1668.

Chan, A.C., Iwashima, M., Turck, C.W., and Weiss, A. (1992). ZAP-70: a 70 kd protein-tyrosine kinase that associates with the TCR zeta chain. Cell *71*, 649–662.

Chemnitz, J.M., Parry, R.V., Nichols, K.E., June, C.H., and Riley, J.L. (2004). SHP-1 and SHP-2 associate with immunoreceptor tyrosine-based switch motif of programmed death 1 upon primary human T cell stimulation, but only receptor ligation prevents T cell activation. J. Immunol. *173*, 945–954.

Coghlan, M.P., Culbert, A.A., Cross, D.A., Corcoran, S.L., Yates, J.W., Pearce, N.J., Rausch, O.L., Murphy, G.J., Carter, P.S., Roxbee Cox, L., et al. (2000). Selective small molecule inhibitors of glycogen synthase kinase-3 modulate glycogen metabolism and gene transcription. Chem. Biol. *7*, 793–803.

Cohen, P., and Frame, S. (2001). The renaissance of GSK3. Nat. Rev. Mol. Cell Biol. *2*, 769–776.

Cohen, P., and Goedert, M. (2004). GSK3 inhibitors: development and therapeutic potential. Nat. Rev. Drug Discov. *3*, 479–487.

Day, C.L., Kaufmann, D.E., Kiepiela, P., Brown, J.A., Moodley, E.S., Reddy, S., Mackey, E.W., Miller, J.D., Leslie, A.J., DePierres, C., et al. (2006). PD-1 expression on HIV-specific T cells is associated with T-cell exhaustion and disease progression. Nature *443*, 350–354.

de Lima, B.D., May, J.S., and Stevenson, P.G. (2004). Murine gammaherpesvirus 68 lacking gp150 shows defective virion release but establishes normal latency in vivo. J. Virol. *78*, 5103–5112.

Dong, H., Zhu, G., Tamada, K., and Chen, L. (1999). B7-H1, a third member of the B7 family, co-stimulates T-cell proliferation and interleukin-10 secretion. Nat. Med. *5*, 1365–1369.

Evans, A., Riva, A., Cooksley, H., Phillips, S., Puranik, S., Nathwani, A., Brett, S., Chokshi, S., and Naoumov, N.V. (2008). Programmed death 1 expression during antiviral treatment of chronic hepatitis B: Impact of hepatitis B e-antigen seroconversion. Hepatology *48*, 759–769.

Frame, S., and Cohen, P. (2001). GSK3 takes centre stage more than 20 years after its discovery. Biochem. J. *359*, 1–16.

Freeman, G.J., Long, A.J., Iwai, Y., Bourque, K., Chernova, T., Nishimura, H., Fitz, L.J., Malenkovich, N., Okazaki, T., Byrne, M.C., et al. (2000). Engagement of the PD-1 immunoinhibitory receptor by a novel B7 family member leads to negative regulation of lymphocyte activation. J. Exp. Med. *192*, 1027–1034.

Freeman, G.J., Wherry, E.J., Ahmed, R., and Sharpe, A.H. (2006). Reinvigorating exhausted HIV-specific T cells via PD-1-PD-1 ligand blockade. J. Exp. Med. *203*, 2223–2227.

Glimcher, L.H. (2007). Trawling for treasure: tales of T-bet. Nat. Immunol. *8*, 448–450.

Grakoui, A., John Wherry, E., Hanson, H.L., Walker, C., and Ahmed, R. (2006). Turning on the off switch: regulation of anti-viral T cell responses in the liver by the PD-1/PD-L1 pathway. J. Hepatol. *45*, 468–472.

Ha, S.J., Mueller, S.N., Wherry, E.J., Barber, D.L., Aubert, R.D., Sharpe, A.H., Freeman, G.J., and Ahmed, R. (2008). Enhancing therapeutic vaccination by blocking PD-1-mediated inhibitory signals during chronic infection. J. Exp. Med. *205*, 543–555.

Harker, J.A., Lewis, G.M., Mack, L., and Zuniga, E.I. (2011). Late interleukin-6 escalates T follicular helper cell responses and controls a chronic viral infection. Science *334*, 825–829.

Harker, J.A., Dolgoter, A., and Zuniga, E.I. (2013). Cell-intrinsic IL-27 and gp130 cytokine receptor signaling regulates virus-specific $CD4^+$ T cell responses and viral control during chronic infection. Immunity *39*, 548–559.

Hodi, F.S., Mihm, M.C., Soiffer, R.J., Haluska, F.G., Butler, M., Seiden, M.V., Davis, T., Henry-Spires, R., MacRae, S., Willman, A., et al. (2003). Biologic activity of cytotoxic T lymphocyte-associated antigen 4 antibody blockade in previously vaccinated metastatic melanoma and ovarian carcinoma patients. Proc. Natl. Acad. Sci. USA *100*, 4712–4717.

Hodi, F.S., O'Day, S.J., McDermott, D.F., Weber, R.W., Sosman, J.A., Haanen, J.B., Gonzalez, R., Robert, C., Schadendorf, D., Hassel, J.C., et al. (2010). Improved survival with ipilimumab in patients with metastatic melanoma. N. Engl. J. Med. *363*, 711–723.

Husain, S.M., Usherwood, E.J., Dyson, H., Coleclough, C., Coppola, M.A., Woodland, D.L., Blackman, M.A., Stewart, J.P., and Sample, J.T. (1999). Murine gammaherpesvirus M2 gene is latency-associated and its protein a target for CD8(+) T lymphocytes. Proc. Natl. Acad. Sci. USA *96*, 7508–7513.

Ishida, M., Iwai, Y., Tanaka, Y., Okazaki, T., Freeman, G.J., Minato, N., and Honjo, T. (2002). Differential expression of PD-L1 and PD-L2, ligands for an inhibitory receptor PD-1, in the cells of lymphohematopoietic tissues. Immunol. Lett. *84*, 57–62.

Kaidanovich-Beilin, O., and Eldar-Finkelman, H. (2006). Peptides targeting protein kinases: strategies and implications. Physiology (Bethesda) *21*, 411–418.

Kanhere, A., Hertweck, A., Bhatia, U., Gökmen, M.R., Perucha, E., Jackson, I., Lord, G.M., and Jenner, R.G. (2012). T-bet and GATA3 orchestrate Th1 and Th2 differentiation through lineage-specific targeting of distal regulatory elements. Nat. Commun. *3*, 1268.

Kao, C., Oestreich, K.J., Paley, M.A., Crawford, A., Angelosanto, J.M., Ali, M.A., Intlekofer, A.M., Boss, J.M., Reiner, S.L., Weinmann, A.S., and Wherry, E.J. (2011). Transcription factor T-bet represses expression of the inhibitory receptor PD-1 and sustains virus-specific CD8+ T cell responses during chronic infection. Nat. Immunol. *12*, 663–671.

Keir, M.E., Freeman, G.J., and Sharpe, A.H. (2007). PD-1 regulates self-reactive CD8+ T cell responses to antigen in lymph nodes and tissues. J. Immunol. *179*, 5064–5070.

Kinter, A.L., Godbout, E.J., McNally, J.P., Sereti, I., Roby, G.A., O'Shea, M.A., and Fauci, A.S. (2008). The common gamma-chain cytokines IL-2, IL-7, IL-15, and IL-21 induce the expression of programmed death-1 and its ligands. J. Immunol. *181*, 6738–6746.

Kroner, A., Mehling, M., Hemmer, B., Rieckmann, P., Toyka, K.V., Mäurer, M., and Wiendl, H. (2005). A PD-1 polymorphism is associated with disease progression in multiple sclerosis. Ann. Neurol. *58*, 50–57.

Latchman, Y., Wood, C.R., Chernova, T., Chaudhary, D., Borde, M., Chernova, I., Iwai, Y., Long, A.J., Brown, J.A., Nunes, R., et al. (2001). PD-L2 is a second ligand for PD-1 and inhibits T cell activation. Nat. Immunol. *2*, 261–268.

Li, C., Li, W., Xiao, J., Jiao, S., Teng, F., Xue, S., Zhang, C., Sheng, C., Leng, Q., Rudd, C.E., et al. (2015). ADAP and SKAP55 deficiency suppresses PD-1 expression in CD8+ cytotoxic T lymphocytes for enhanced anti-tumor immunotherapy. EMBO Mol. Med. *7*, 754–769.

Lu, P., Youngblood, B.A., Austin, J.W., Mohammed, A.U., Butler, R., Ahmed, R., and Boss, J.M. (2014). Blimp-1 represses CD8 T cell expression of PD-1 using a feed-forward transcriptional circuit during acute viral infection. J. Exp. Med. *211*, 515–527.

Macián, F., López-Rodríguez, C., and Rao, A. (2001). Partners in transcription: NFAT and AP-1. Oncogene *20*, 2476–2489.

Martinez, G.J., Pereira, R.M., Äijö, T., Kim, E.Y., Marangoni, F., Pipkin, M.E., Togher, S., Heissmeyer, V., Zhang, Y.C., Crotty, S., et al. (2015). The transcription factor NFAT promotes exhaustion of activated $CD8^+$ T cells. Immunity *42*, 265–278.

Masuda, E.S., Imamura, R., Amasaki, Y., Arai, K., and Arai, N. (1998). Signalling into the T-cell nucleus: NFAT regulation. Cell. Signal. *10*, 599–611.

Mathieu, M., Cotta-Grand, N., Daudelin, J.F., Thébault, P., and Labrecque, N. (2013). Notch signaling regulates PD-1 expression during CD8(+) T-cell activation. Immunol. Cell Biol. *91*, 82–88.

McDermott, D.S., and Varga, S.M. (2011). Quantifying antigen-specific CD4 T cells during a viral infection: CD4 T cell responses are larger than we think. J. Immunol. *187*, 5568–5576.

Milho, R., Smith, C.M., Marques, S., Alenquer, M., May, J.S., Gillet, L., Gaspar, M., Efstathiou, S., Simas, J.P., and Stevenson, P.G. (2009). In vivo imaging of murid herpesvirus-4 infection. J. Gen. Virol. *90*, 21–32.

Neal, J.W., and Clipstone, N.A. (2001). Glycogen synthase kinase-3 inhibits the DNA binding activity of NFATc. J. Biol. Chem. *276*, 3666–3673.

Nishimura, H., Okazaki, T., Tanaka, Y., Nakatani, K., Hara, M., Matsumori, A., Sasayama, S., Mizoguchi, A., Hiai, H., Minato, N., and Honjo, T. (2001). Autoimmune dilated cardiomyopathy in PD-1 receptor-deficient mice. Science *291*, 319–322.

Oestreich, K.J., Yoon, H., Ahmed, R., and Boss, J.M. (2008). NFATc1 regulates PD-1 expression upon T cell activation. J. Immunol. *181*, 4832–4839.

Ohteki, T., Parsons, M., Zakarian, A., Jones, R.G., Nguyen, L.T., Woodgett, J.R., and Ohashi, P.S. (2000). Negative regulation of T cell proliferation and interleukin 2 production by the serine threonine kinase GSK-3. J. Exp. Med. *192*, 99–104.

Okazaki, T., Maeda, A., Nishimura, H., Kurosaki, T., and Honjo, T. (2001). PD-1 immunoreceptor inhibits B cell receptor-mediated signaling by recruiting src homology 2-domain-containing tyrosine phosphatase 2 to phosphotyrosine. Proc. Natl. Acad. Sci. USA *98*, 13866–13871.

Okazaki, T., Iwai, Y., and Honjo, T. (2002). New regulatory co-receptors: inducible co-stimulator and PD-1. Curr. Opin. Immunol. *14*, 779–782.

Palmer, B.E., Neff, C.P., Lecureux, J., Ehler, A., Dsouza, M., Remling-Mulder, L., Korman, A.J., Fontenot, A.P., and Akkina, R. (2013). In vivo blockade of the PD-1 receptor suppresses HIV-1 viral loads and improves CD4+ T cell levels in humanized mice. J. Immunol. *190*, 211–219.

Parry, R.V., Chemnitz, J.M., Frauwirth, K.A., Lanfranco, A.R., Braunstein, I., Kobayashi, S.V., Linsley, P.S., Thompson, C.B., and Riley, J.L. (2005). CTLA-4 and PD-1 receptors inhibit T-cell activation by distinct mechanisms. Mol. Cell. Biol. *25*, 9543–9553.

Prasad, K.V., Cai, Y.C., Raab, M., Duckworth, B., Cantley, L., Shoelson, S.E., and Rudd, C.E. (1994). T-cell antigen CD28 interacts with the lipid kinase phosphatidylinositol 3-kinase by a cytoplasmic Tyr(P)-Met-Xaa-Met motif. Proc. Natl. Acad. Sci. USA *91*, 2834–2838.

Quigley, M., Pereyra, F., Nilsson, B., Porichis, F., Fonseca, C., Eichbaum, Q., Julg, B., Jesneck, J.L., Brosnahan, K., Imam, S., et al. (2010). Transcriptional analysis of HIV-specific CD8+ T cells shows that PD-1 inhibits T cell function by upregulating BATF. Nat. Med. *16*, 1147–1151.

Raab, M., Wang, H., Lu, Y., Smith, X., Wu, Z., Strebhardt, K., Ladbury, J.E., and Rudd, C.E. (2010). T cell receptor "inside-out" pathway via signaling module SKAP1-RapL regulates T cell motility and interactions in lymph nodes. Immunity *32*, 541–556.

Rudd, C.E. (1999). Adaptors and molecular scaffolds in immune cell signaling. Cell *96*, 5–8.

Rudd, C.E., and Schneider, H. (2003). Unifying concepts in CD28, ICOS and CTLA4 co-receptor signalling. Nat. Rev. Immunol. *3*, 544–556.

Rudd, C.E., Trevillyan, J.M., Dasgupta, J.D., Wong, L.L., and Schlossman, S.F. (1988). The CD4 receptor is complexed in detergent lysates to a protein-tyrosine kinase (pp58) from human T lymphocytes. Proc. Natl. Acad. Sci. USA *85*, 5190–5194.

Samelson, L.E. (2002). Signal transduction mediated by the T cell antigen receptor: the role of adapter proteins. Annu. Rev. Immunol. *20*, 371–394.

Schroeder, J.H., Bell, L.S., Janas, M.L., and Turner, M. (2013). Pharmacological inhibition of glycogen synthase kinase 3 regulates T cell development in vitro. PLoS ONE *8*, e58501.

Sharpe, A.H., and Freeman, G.J. (2002). The B7-CD28 superfamily. Nat. Rev. Immunol. *2*, 116–126.

Sharpe, A.H., Wherry, E.J., Ahmed, R., and Freeman, G.J. (2007). The function of programmed cell death 1 and its ligands in regulating autoimmunity and infection. Nat. Immunol. *8*, 239–245.

Smith, X., Schneider, H., Köhler, K., Liu, H., Lu, Y., and Rudd, C.E. (2013). The chemokine CXCL12 generates costimulatory signals in T cells to enhance phosphorylation and clustering of the adaptor protein SLP-76. Sci. Signal. *6*, ra65.

Song, M., Park, J.E., Park, S.G., Lee, H., Choi, H.K., Park, B.C., Ryu, S.E., Kim, J.H., and Cho, S. (2009). NSC-87877, inhibitor of SHP-1/2 PTPs, inhibits dual-specificity phosphatase 26 (DUSP26). Biochem. Biophys. Res. Commun. *381*, 491–495.

Srinivasan, M., and Frauwirth, K.A. (2007). Reciprocal NFAT1 and NFAT2 nuclear localization in CD8+ anergic T cells is regulated by suboptimal calcium signaling. J. Immunol. *179*, 3734–3741.

Staron, M.M., Gray, S.M., Marshall, H.D., Parish, I.A., Chen, J.H., Perry, C.J., Cui, G., Li, M.O., and Kaech, S.M. (2014). The transcription factor FoxO1 sustains expression of the inhibitory receptor PD-1 and survival of antiviral CD8(+) T cells during chronic infection. Immunity *41*, 802–814.

Stevenson, P.G., Belz, G.T., Castrucci, M.R., Altman, J.D., and Doherty, P.C. (1999). A gamma-herpesvirus sneaks through a CD8(+) T cell response primed to a lytic-phase epitope. Proc. Natl. Acad. Sci. USA *96*, 9281–9286.

Takebe, Y., Seiki, M., Fujisawa, J., Hoy, P., Yokota, K., Arai, K., Yoshida, M., and Arai, N. (1988). SR alpha promoter: an efficient and versatile mammalian cDNA expression system composed of the simian virus 40 early promoter and the R-U5 segment of human T-cell leukemia virus type 1 long terminal repeat. Mol. Cell. Biol. *8*, 466–472.

Topalian, S.L., Drake, C.G., and Pardoll, D.M. (2015). Immune checkpoint blockade: a common denominator approach to cancer therapy. Cancer Cell *27*, 450–461.

Tseng, S.Y., Otsuji, M., Gorski, K., Huang, X., Slansky, J.E., Pai, S.I., Shalabi, A., Shin, T., Pardoll, D.M., and Tsuchiya, H. (2001). B7-DC, a new dendritic cell molecule with potent costimulatory properties for T cells. J. Exp. Med. *193*, 839–846.

Valk, E., Leung, R., Kang, H., Kaneko, K., Rudd, C.E., and Schneider, H. (2006). T cell receptor-interacting molecule acts as a chaperone to modulate surface expression of the CTLA-4 coreceptor. Immunity *25*, 807–821.

Veale, M., Raab, M., Li, Z., da Silva, A.J., Kraeft, S.K., Weremowicz, S., Morton, C.C., and Rudd, C.E. (1999). Novel isoform of lymphoid adaptor FYN-T-binding protein (FYB-130) interacts with SLP-76 and up-regulates interleukin 2 production. J. Biol. Chem. *274*, 28427–28435.

Veillette, A., Bookman, M.A., Horak, E.M., Samelson, L.E., and Bolen, J.B. (1989). Signal transduction through the CD4 receptor involves the activation of the internal membrane tyrosine-protein kinase p56lck. Nature *338*, 257–259.

Velu, V., Titanji, K., Zhu, B., Husain, S., Pladevega, A., Lai, L., Vanderford, T.H., Chennareddi, L., Silvestri, G., Freeman, G.J., et al. (2009). Enhancing SIV-specific immunity in vivo by PD-1 blockade. Nature *458*, 206–210.

Virgin, H.W., Wherry, E.J., and Ahmed, R. (2009). Redefining chronic viral infection. Cell *138*, 30–50.

Wacker, M.J., and Godard, M.P. (2005). Analysis of one-step and two-step real-time RT-PCR using SuperScript III. J. Biomol. Tech. *16*, 266–271.

Weiss, A., and Littman, D.R. (1994). Signal transduction by lymphocyte antigen receptors. Cell *76*, 263–274.

West, E.E., Jin, H.T., Rasheed, A.U., Penaloza-Macmaster, P., Ha, S.J., Tan, W.G., Youngblood, B., Freeman, G.J., Smith, K.A., and Ahmed, R. (2013). PD-L1 blockade synergizes with IL-2 therapy in reinvigorating exhausted T cells. J. Clin. Invest. *123*, 2604–2615.

Wherry, E.J. (2011). T cell exhaustion. Nat. Immunol. *12*, 492–499.

Wherry, E.J., Ha, S.J., Kaech, S.M., Haining, W.N., Sarkar, S., Kalia, V., Subramaniam, S., Blattman, J.N., Barber, D.L., and Ahmed, R. (2007). Molecular signature of CD8+ T cell exhaustion during chronic viral infection. Immunity *27*, 670–684.

Wolchok, J.D., Kluger, H., Callahan, M.K., Postow, M.A., Rizvi, N.A., Lesokhin, A.M., Segal, N.H., Ariyan, C.E., Gordon, R.A., Reed, K., et al. (2013). Nivolumab plus ipilimumab in advanced melanoma. N. Engl. J. Med. *369*, 122–133.

Wood, J.E., Schneider, H., and Rudd, C.E. (2006). TcR and TcR-CD28 engagement of protein kinase B (PKB/AKT) and glycogen synthase kinase-3 (GSK-3) operates independently of guanine nucleotide exchange factor VAV-1. J. Biol. Chem. *281*, 32385–32394.

Woodgett, J.R. (1990). Molecular cloning and expression of glycogen synthase kinase-3/factor A. EMBO J. *9*, 2431–2438.

Xiao, G., Deng, A., Liu, H., Ge, G., and Liu, X. (2012). Activator protein 1 suppresses antitumor T-cell function via the induction of programmed death 1. Proc. Natl. Acad. Sci. USA *109*, 15419–15424.

Yokosuka, T., Takamatsu, M., Kobayashi-Imanishi, W., Hashimoto-Tane, A., Azuma, M., and Saito, T. (2012). Programmed cell death 1 forms negative costimulatory microclusters that directly inhibit T cell receptor signaling by recruiting phosphatase SHP2. J. Exp. Med. *209*, 1201–1217.

Zinselmeyer, B.H., Heydari, S., Sacristán, C., Nayak, D., Cammer, M., Herz, J., Cheng, X., Davis, S.J., Dustin, M.L., and McGavern, D.B. (2013). PD-1 promotes immune exhaustion by inducing antiviral T cell motility paralysis. J. Exp. Med. *210*, 757–774.

Immunity, Volume *44*

Supplemental Information

Glycogen Synthase Kinase 3 Inactivation Drives T-bet-Mediated Downregulation of Co-receptor PD-1 to Enhance CD8$^+$ Cytolytic T Cell Responses

Alison Taylor, James A. Harker, Kittiphat Chanthong, Philip G. Stevenson, Elina I. Zuniga, and Christopher E. Rudd

Figure S1

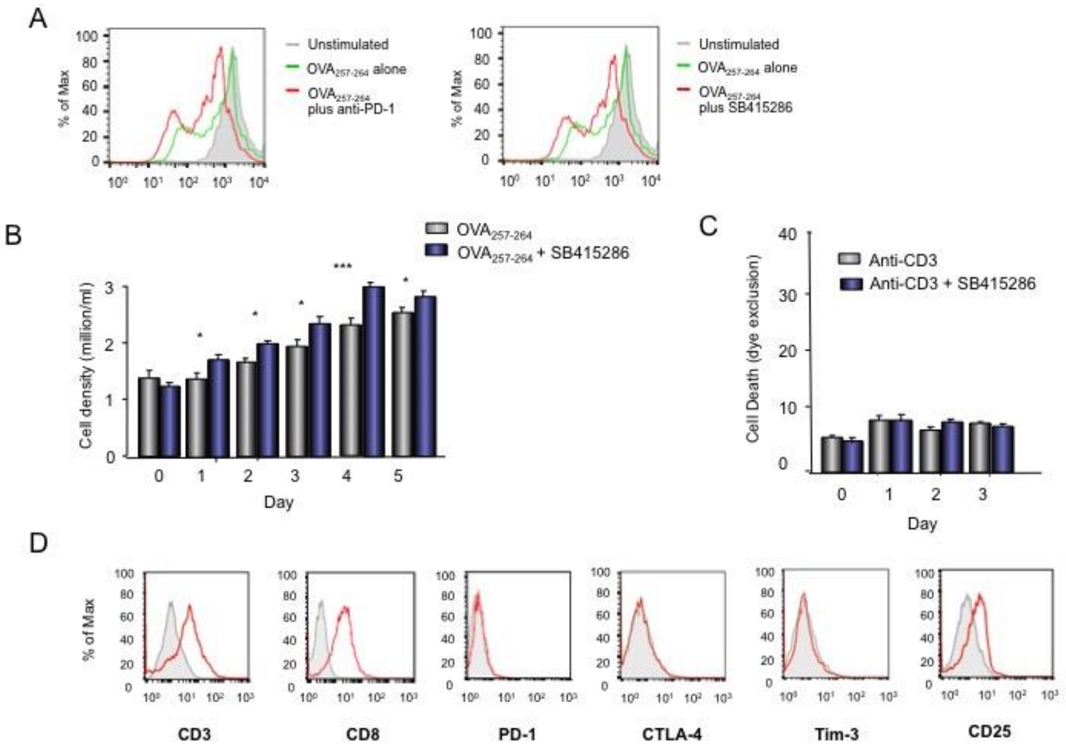

Figure S1, related to Figure 1. GSK-3 inhibition increased T-cell proliferation and cell number.

Flow cytometry profiles of OT-1 T-cells activated by EL-4-OVA in the presence or absence of anti-PD-1 or SB415286. OT-1 cells were labelled with CFSE prior to culturing with mitomycin C treated EL-4-OVA cells in the presence or absence of SB415286 or anti-PD1 for 5 days prior to analysis by FACs. Left panel: Profile in the presence or absence of anti-PD-1. Right panel: Profile in the presence or absence of anti-PD-1. The addition of SB415286 enhances this proliferation (red line).

(A) Histogram showing an increase in OT-1 cell number in the presence of SB415286. OT-1 cells were cultured with mitomycin C treated EL-4-OVA cells in the presence or absence of SB415286 or anti-PD1 for 5 days prior to analysis by FACs.

(B) Histogram showing no effect of SB415286 on T-cell viability in response to anti-CD3. OT-1 cells were cultured in the presence of soluble anti-CD3 (2C11) and SB415286

(10μM) for various days. Aliquots of cells were then measured for viability using trypan blue inclusion.

(C) FACs profiles resting OT-1 T-cells showing lack of expression of PD-1, CTLA-4 and Tim-3. Cells were positive for CD3 and CD8.

Figure S2

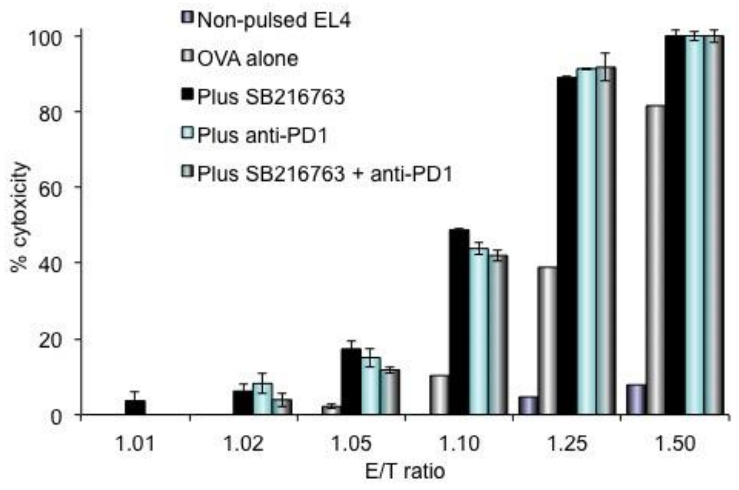

Figure S2, related to Figure 2. GSK-3 potentiates OT-1 cytolytic killing of EL4-OVA target cells by down-regulating PD-1. Related to Figure 2.

As in Figure 2 except that the figure shows a measurement of % target killing of EL4-OVA targets by OT-1 CD8+ CTL incubated in the presence or absence of GK-3 inhibitor SB216763 with or without blocking anti-PD-1.

Figure S3

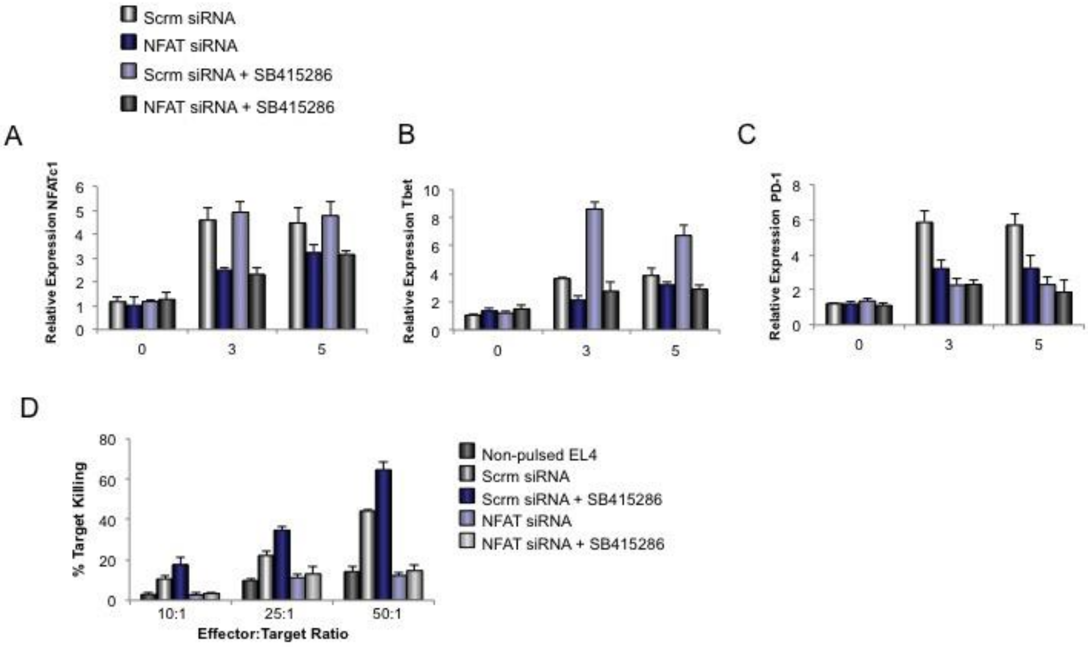

Figure S3, related to Figure 3. siRNA Knockdown of NFAT inhibits Tbet, PD-1 transcription and CTL function. OT-1 cells were transfected with either scrambled or NFATc1 siRNA prior to culturing with EL-4-OVA in the presence or absence of SB415286 and assessed for NFATc1 transcription at days 0, 3 and 5 or CTL killing at day 5.

(A) NFATc1 siRNA reduced levels of detectable NFATc1 transcriptions.

(B) NFATc1 siRNA reduced levels of Tbet transcription in the absence or presence of SB415286.

(C) NFATc1 siRNA reduced levels of PD-1 transcription in the absence or presence of SB415286.

(D) NFATc1 siRNA inhibited CTL function when compared to scrambled control siRNA transfected cells.

Figure S4

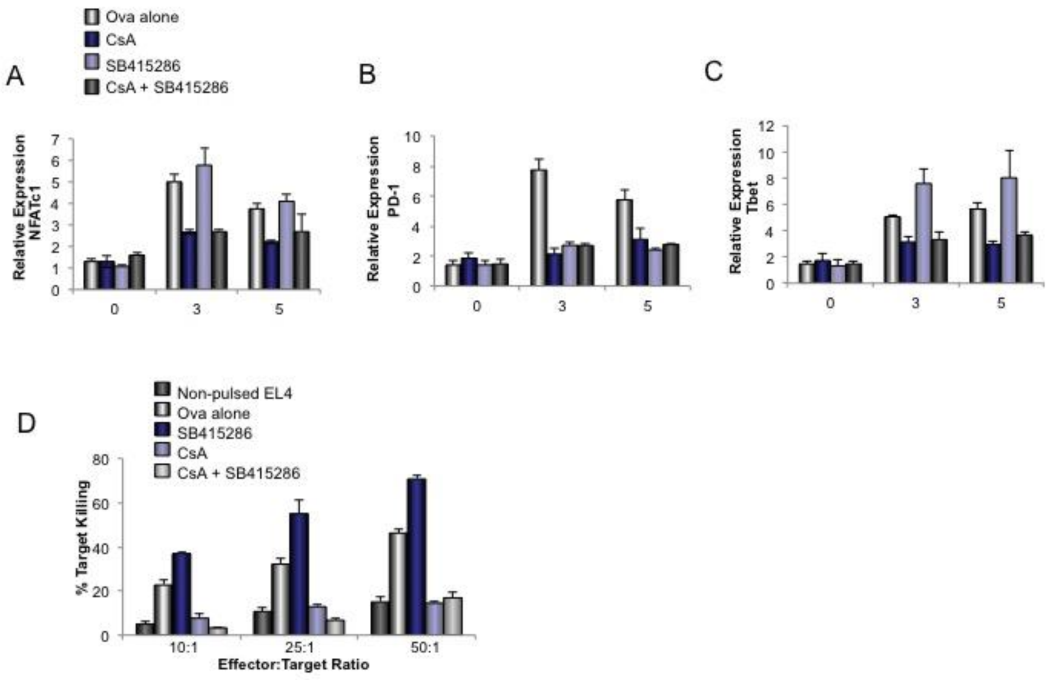

Figure S4, related to Figure 3. Inhibition of NFAT by Cyclosporin A (CsA) reduces PD-1 and Tbet expression and cytolytic activity. OT-1 cells cultured with EL-4-OVA and cyclosporine A (CsA) in the presence or absence of SB415286 and assessed for transcription at days 0, 3 and 5 or CTL killing at day 5.

(A) CsA reduced levels of detectable NFATc1 transcriptions.

(B) CsA reduced Tbet transcription in the absence or presence of SB415286.

(C) CsA reduced PD-1 transcription in the absence or presence of SB415286.

(D) CsA siRNA inhibited CTL function when compared to scrambled control siRNA transfected cells.

Figure S5

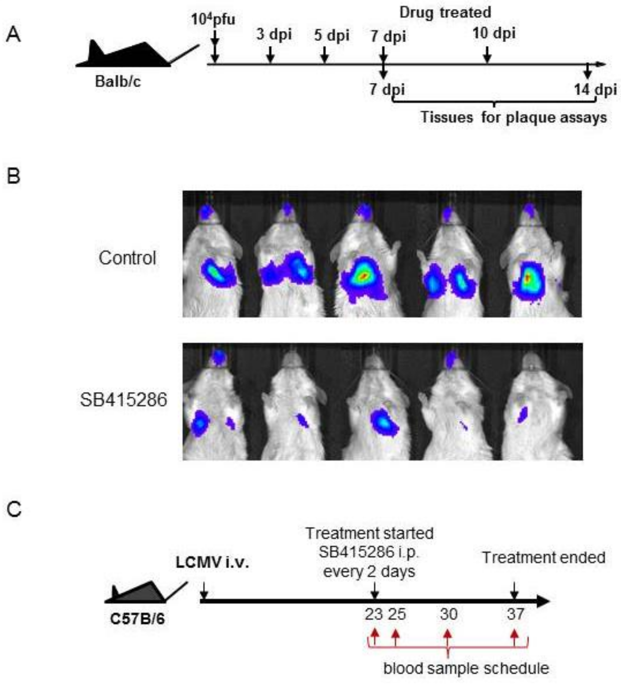

Figure S5, related to Figures 5 and 6. *In vivo* GSK-3 inhibition increased acute MHV-68 viral clearance via PD-1 down-regulation. Balb/c mice were intra-nasally infected with MHV-68 with/without an intra-peritoneal injection of SB415286. **(A)** Depicts scheme of injection regime in Figure 5. **(B)** Images from luciferase imaging of mice on 7-day post-infection (upper panel) non-treated; (lower panel) SB415286 in Figure 5. (**C**) Depicts scheme of injection regime for LCMV-Cl 13 model in Figure 6.

EXTENDED EXPERIMENTAL PROCEDURES

Mice

C57BL/6 and BALB/c (Harlan UK Ltd) were housed at the Department of Pathology and the Central Biological Services (Cambridge University). Mice infected when 6–8 weeks old under the Home Office Project Licence 80/2189 (obtained from The Jackson Laboratory).

Antibodies/Reagents

Anti-CD3 (2C11; BioXCell), anti-PD-1 (CD279, J43; BioXCell), anti-CTLA-4 (9H10 BioXCell) and PD-L1 (E1L3N; Cell Signaling Technology), anti-Granzyme B (GMZB; ab 17929; Abcam plc), anti-T-bet / Tbx21 (ab53174; Abcam plc) and anti-GSK-3α/β (Biolegend) were purchased commercially. Similarly, various antibodies to CD44, CD62L, Tim3, BTLA, NKG2D, CD122, IL-2R alpha, CD25, FasL and CD8 were purchased from Cell Signaling Technology, Abcam plc and Biolegend as directly conjugated antibodies. Peptide 10nM $OVA_{257-264}$ was purchased from Bachem, while biotinylated D^b GP_{33-41} monomers were provide by the NIH tetramer facility and APC labelled tetramers generated using Streptavidin-APC (Invitrogen). siRNAs for GSK-3α/β, GSK-3α and Tbet and control scrambled siRNAs were synthesized by Cell Signaling Technology.

GSK3 inhibitors were purchased from various sources including SB415286 3-(3-chloro-4-hydroxyphenylamino)-4-(2-nitrophenyl)-1H-pyrrole-2,5-dione and SB216763 [3-(2,4-dichlorophenyl)-4-(1-methyl-1H-indol-3-yl)-1H-pyrrole-2,5-dione] from Abcam plc; CHIR-99021 (CT99021) [6-(2-(4-(2,4-dichlorophenyl)-5-(4-methyl-1H-imidazol-2-yl)pyrimidin-2-ylamino)ethylamino)nicotinonitrile hydrochloride] and L803-mts from T*ocris* Bioscience *Boston* Biochem, R & D systems.

Cell Culture and Nuclear Transfection

Primary mouse T cells (OT-1, C57/b6) were isolated from spleens and cultured *in vitro* in RPMI 1640 medium supplemented with 10% FCS, 50 μM β-mercaptoethanol, 2 mM L-glutamine, 100 U/ml penicillin and streptomycin, (GIBCO)(Raab et al., 2010; Veale et al., 1999). Spleen cells were treated with a hypotonic buffer with 0.15M NH4CL, 10mM KHCO3 and 0.1mM EDTA, pH 7.2 to eliminate red blood cells. A T cell enriched population was purified by use of T cell purification columns (R&D Systems, Minneapolis, MN). OVA specific CD8+ cytoytic T-cells were generated by incubating isolated splenocytes OT-1 mice with SIINFEKL peptide of OVA ($OVA_{257-264}$) at 10ng/mL for 5-7 days. Alternatively, the thymoma EL-4 cell line was used to present $OVA_{257-264}$ to primary T-cells. EL-4 cells were incubated with 10nM $OVA_{257-264}$ peptide (Bachem) for 1 hour at 37°C and treated with mytomycin C (Sigma-Aldrich, St. Louis, MO) (final concentration of 10μg/mL) prior to mixing with primary T-cells by co-culturing at a ratio of 1:5 of EL4 and T cells in order to generate cytotoxic T cells.

CTLs were generated in the presence or absence of inhibitors and/or anti-PD-1 or anti-PDL-1 blockade for 5-7 days prior to washing and analysis by FACs, PCR or in cytoxicity assays. In some cases, naïve OT-1 T cells were first incubated with OVA peptide with or without GSK-3 inhibitors and/or anti-PD-1 or PD-L1-Fc (1-3ug/ml). All compounds were suspended in DMSO to give a stock solution of 25mM and diluted to a concentration of 1-10uM *in vitro*. NSC 87877 (inhibitor of SHP1/2 PTPs and dual-specificity phosphatase 26 (DUSP26), used as a concentration of 50μM (T*ocris* Bioscience *Boston* Biochem) (Song et al., 2009) and cyclosporin A (CsA) (Sigma) at 0.5ug/ml.

In certain cases, naïve cells were subjected to nuclear transfection in the presence of various siRNA oligos (i.e. GSK-3). 3.0-5.0ug of siRNAs were added to 1 x 10^6 T-cells and suspended in 100ul of Nucleofector™ solution for T cells (Amaxa Biosystems, Cologne, Germany). Cells and oligos were then transferred into a cuvette and electroporated using program X-01 of the Nucleofector™ (Amaxa

Biosystems), and then immediately transferred into pre-warmed RPMI medium, as previously described(Smith et al., 2013; Valk et al., 2006). In certain instances, pre-activated T-cells were transfected with mouse PD-1 in an SR alpha promoter (pPD1) composed of the simian virus 40 early promoter and the R-U5 segment of human T-cell leukemia virus type 1 long terminal repeat (Takebe et al., 1988).

Cytotoxicity Assays

For *in vitro* cytotoxic assays, transfected T cells were plated in 96-well plates at the start of culture with activating EL4 cells pulsed with OVA peptide except in the case of the MHV-68 in which EL4s were pulsed with the M2 peptide ($M2_{91–99}$, GFNKLRSTL) (SIGMA-Genosys, Haverhill, UK) (Husain et al., 1999). Cytotoxicity was assayed using a Cytotox 96 nonradioactive kit (Promega) following the instructions provided. In brief, purified T cells were plated in 96-well plates at the effector/target ratios shown using 10^4 EL4 (OVA peptide-pulsed, except in the case of the MHV-68 in which EL4s were pulsed with the M2 peptide. Target cells per well were in a final volume of 200 µl per well using RPMI lacking phenol red. Lactate dehydrogenase release was assayed after 4 h incubation at 37°C by removal of 50 µl supernatant from each well and incubation with substrate provided for 30 min and the absorbance read at 490 nm using the Thermomax plate reader (Molecular Devices). Percentage cytotoxicity = ((experimental $effector_{spontaneous}$ – target spontaneous)/($target_{maximum}$ – target spontaneous)) x 100. All cytotoxicity assays were reproducible in at least three independent assays (Jenkins, MR *et al*, 2009). Scatchard analysis was conducted based on the ratio of dead target cells to live effector cells versus the total number of targets killed. Linearized data obtained first from a non-linear curve displaying E/T ratios in order to determine Kd and BMax values using GraphPad Prism software.

Quantitative real-time PCR

Single-strand cDNA was synthesized with an RT-PCR kit (Qiagen, Hilden, Germany) according to the manufacturer's instructions. Reverse transcription was performed using the RNA polymerase chain reaction (PCR) core kit (Applied Biosystems). Quantitative real-time PCR used SYBR green technology (Roche) on cDNA generated from the reverse transcription of purified RNA. After preamplification (95°C for 2 min), the PCRs were amplified for 40 cycles (95°C for 15 s and 60°C for 60 s) in a sequence detection system (PE Prism 7000; Perkin-Elmer Applied Biosystems, USA). The exponential phase, linear phase and plateau phase of PCR amplification were carefully monitored to ensure a measurement of real time transcription (Wacker and Godard, 2005). mRNA expression was normalized against GAPDH expression using the standard curve method. PD-1-FW, 5-CCGCCTTCTGTAATGGTTTGA-3; PD-1-RV, 5-GGGCAGCTGTATGATCTGGAA-3; Tbet-FW, 5-GATCGTCCTGCAGTCTCTCC-3; Tbet-RW, 5-AACTGTGTTCCCGAGGT GTC-3; GAPDH-FW, 5-CAACAGCAACTCCCACTCTTC-3; GAPDH- RW, 5-GGTCCAGGGTT TCTTACTCCTT-3. NFATc1 siRNA was purchased from Santa Cruz (http://www.scbt.com/datasheet-36054-nfatc1-sirna-m.html).

Chromatin immunoprecipitation (ChIP) assay

C57/b6 primary T cells were purified using CD3+ T cell enrichment columns, 3 x 10^6 cells were used as a resting control while remaining cells were cultured with anti-CD3 with/without SB415286 for 72h. Chromatin was prepared from all samples (3 x 10^6 cells) and used for ChIP assay following the manufacturers protocols - Pierce™ Agarose ChIP kit (Thermo scientific #26156). The immunoprecipitation step was performed independently using one of two T-bet antibodies (H-210 or 4B10, Santa cruz) or a positive control –anti-RNA polymerase II, or negative control Rabbit Ig, both of the latter being provided in the kit. The resulting purified DNA with bound

Ab was then analysed using quantitative RT-PCR. CHiP assay was also performed using Jurkat cell line 3×10^6 cells with/without SB415286 for 72 hrs

Luciferase reporter assay

C57/b6 primary T cells were purified using CD3+ T cell enrichment columns and cultured with anti-CD3 with/without SB415286 for 72h. Cells were washed and transfected with a promoter *Ifng CNS-12* construct driven only by a Tbet element (kind gift of Dr. Graham Lord, King's College), and left to rest overnight. 18h post transfection, cells were incubated in anti-CD3 coated plates (2×10^5/well) for 6h at 37^0C. Plates were washed gently with PBS twice and cells re-suspended in 20ul of Passive lysis buffer (Dual-luciferase® Reporter Assay System (Promega)). Following 20 min incubation on rocking platform, plates incubated at -20^0C overnight. Thawed samples transferred to FACs tubes and 50ul of luciferase substrate added before measuring with luminometer, 50ul of Renilla added to each tube and re-measured. Calculations performed using (luciferase measurement/renilla measurement) x 1000.

***In vivo* Priming OT-I Tg cells**

OVA peptide (1μg) was injected intravenously into OT-I Tg mice with and without SB415286 (10μg) in 100μl of PBS. Spleens were harvested after 7 days and T cells purified. Longer experiments utilized a repeat injection on day 7 that was reminiscent of the initial injection. Spleens were then harvested on day 14 and T cells purified.

Viruses and Luciferase imaging in vivo

Luciferase tagged- MHV-68 viral stocks were prepared as described (de Lima et al., 2004; Gillet et al., 2006). BALB/c mice were infected with luciferase expressing MHV-68 (10^4 p.f.u.) intranasally under general anaesthesia when 6–12 weeks old.

Intranasal infections with anaesthesia were in 30 μl aliquots. Mice underwent a treatment regime as depicted on days 0, 3, 5, 7 and 10 of either PBS or SB415286 100μl intraperitoneally. All experiments conformed to local animal ethics regulations; those in Cambridge also followed Home Office Project Licence 80/2189. For luciferase imaging, mice were injected intraperitoneally with luciferin (2 ug per mouse), anaesthetized with isoflurane and scanned with an IVIS Lumina (Caliper Life Sciences) as previously described (Milho et al., 2009). SB415286 was dosed at 10μg/kg based on a pilot dose response study while anti-PD1/PD-L1 treatment was conducted at 100ug per dose/mouse *in vivo*.

For quantitative comparisons, we used Living Image software (Caliper Life Sciences) to obtain the maximum radiance (photons per s per cm^2 per steradian, i.e. photons s^{-1} cm^{-2} sr^{-1}) over each region of interest, relative to a negative control region. For viral infectivity assays, MHV-68 stocks were titrated by plaque assay on BHK-21 cells (de Lima et al., 2004). Cell monolayers were incubated with virus (2 h, 37 °C), overlaid with 0.3% carboxymethylcellulose, and 4 days later fixed and stained for plaque counting. Infectious virus in lungs was measured by freeze–thawing the lungs and homogenizing them in 1 ml complete medium prior to plaque assay. Latent virus was measured by infectious centre assay(de Lima et al., 2004): spleen cells were co-cultured with BHK-21 cells, then fixed and stained for plaque counting after 4 days. Plaque assay titres of freeze–thawed lymphoid homogenates were always <1% of infectious centre assay titres, so the latter essentially measured reactivated latent virus.

For LCMV, LCMV-Arm and Cl 13 strains were prepared as described (Harker et al., 2013; Harker et al., 2011). Viruses were also grown, identified, and quantified as described (Ahmed et al., 1984; Borrow et al., 1995). 6- to 8-week-old mice were infected intravenously (i.v.) with 2×10^6 pfu of LCMV-Arm or Cl 13. Mice received doses of SB415286 or PBS every 48h from day 23 through until day 37. LCMV titers

in the serum were determined by vero cell plaque assays as described previously[53.] Assay vero cells (ATCC) were seeded in 96 well plates and incubated with serial dilutions of serum or tissue homogenate for 20 hr. Cells were fixed with 1% paraformaldehyde, blocked with PBS containing 10% FBS for 1 hr, and then incubated with supernatant from 113 hybridoma cells (kindly provided by M.B. Oldstone, TSRI). Finally, the cells were incubated with goat anti-mouse IgG conjugated with Cy3 (Jackson Immunoresearch) and foci were counted by fluorescent microscope. LCMV-specific ELISAs and avidity assays were done as previously described (Harker et al., 2011).

Statistical Analysis

Unpaired Student's t tests or ANOVA tests were performed using the InStat 3.0 software (GraphPad, CA). In certain instances, statistics were done using 2-way ANOVA, or by non-parametric Mann Whitney at each timepoint. * $P < 0.05$, ** $P < 0.01$, *** $P < 0.001$.

References

Ahmed, R., Salmi, A., Butler, L.D., Chiller, J.M., and Oldstone, M.B. (1984). Selection of genetic variants of lymphocytic choriomeningitis virus in spleens of persistently infected mice. Role in suppression of cytotoxic T lymphocyte response and viral persistence. J. Exp. Med. *160*, 521

Borrow, P., Evans, C.F., and Oldstone, M.B. (1995). Virus-induced immunosuppression: immune system-mediated destruction of virus-infected dendritic cells results in generalized immune suppression. Journal of virology *69*, 1059-1070.

de Lima, B.D., May, J.S., and Stevenson, P.G. (2004). Murine gammaherpesvirus 68 lacking gp150 shows defective virion release but establishes normal latency in vivo. Journal of virology *78*, 5103-5112.

Gillet L, Gill MB, Colaco S, Smith CM, Stevenson PG. (2006). Murine gammaherpesvirus-68 glycoprotein B presents a difficult neutralization target to
